# An Audit of Pre-Pregnancy Maternal Obesity and Diabetes Screening in Rural Regional Tasmania and Its Impact on Pregnancy and Neonatal Outcomes

**DOI:** 10.3390/ijerph182212006

**Published:** 2021-11-16

**Authors:** Sharon P. Luccisano, Heinrich C. Weber, Giuliana O. Murfet, Iain K. Robertson, Sarah J. Prior, Andrew P. Hills

**Affiliations:** 1 Tasmanian Health Service—North West, Brickport Road, Burnie, TAS 7320, Australia; heinrich.weber@ths.tas.gov.au (H.C.W.); Giuliana.murfet@ths.tas.gov.au (G.O.M.); 2 Clifford Craig Foundation, Launceston, TAS 7250, Australia; iain.robertson@utas.edu.au; 3 School of Health Sciences, Newnham Campus, University of Tasmania, Launceston, TAS 7250, Australia; andrew.hills@utas.edu.au; 4 School of Medicine, Cradle Coast Campus, University of Tasmania, 16-20 Mooreville Road, Burnie, TAS 7320, Australia; sarah.prior@utas.edu.au

**Keywords:** adverse maternal outcomes, gestational diabetes, gestational weight gain, obesity, obstetric complications, pregnancy

## Abstract

Maternal obesity in pregnancy, a growing health problem in Australia, adversely affects both mothers and their offspring. Gestational diabetes mellitus (GDM) is similarly associated with adverse pregnancy and neonatal complications. A low-risk digital medical record audit of antenatal and postnatal data of 2132 pregnant mothers who gave birth between 2016–2018 residing in rural-regional Tasmania was undertaken. An expert advisory group guided the research and informed data collection. Fifty five percent of pregnant mothers were overweight or obese, 43.6% gained above the recommended standards for gestational weight gain and 35.8% did not have an oral glucose tolerance test. The audit identified a high prevalence of obesity among pregnant women and low screening rates for gestational diabetes mellitus associated with adverse maternal and neonatal pregnancy outcomes. We conclude that there is a high prevalence of overweight and obesity among pregnant women in rural regional Tasmania. Further GDM screening rates are low, which require addressing.

## 1. Introduction

The growing obesity pandemic is impacting pregnancies resulting in adverse outcomes in both mother and neonate. Australia is at the forefront of this obesity pandemic and 55% of the adult population live with overweight or obesity, with higher rates reported in rural areas [1,2]. Between 2016 and 2018, in a Tasmanian outer regional rural area, approximately half of pregnant mothers were living with overweight or obesity [3]. Obesity during pregnancy is associated with increased risk of gestational diabetes mellitus (GDM), pre-eclampsia, macrosomia, neonatal hypoglycaemia, instrumental and caesarean deliveries, post-partum haemorrhage (PPH) and neonatal death [4,5].

Women living with obesity have a substantially higher risk of developing GDM than women with a healthy weight BMI 18.5 to 25 kg/m^2^ [2,4,6]. GDM, defined as glucose intolerance during pregnancy, is the most common medical complication of pregnancy. Women diagnosed with GDM are offered specialised care and provided education, support, and early intervention to maintain target glycaemic levels. Offspring of women with above target glycaemic levels are at higher risk of diabetes-related complications, including neonatal macrosomia and hypoglycaemia. Other predictors of neonatal macrosomia (birth weight > 4000 g) include a high pre-pregnancy BMI, excessive GWG and prolonged gestation [7]. Furthermore, pregnant women with suboptimal GDM management and a higher BMI have a greater risk of hypertensive disorders during pregnancy, including preeclampsia [4,7,8,9].

GDM is typically diagnosed between 24- and 28-weeks’ gestation via a 75 g oral glucose tolerance test (OGTT). Early screening is recommended for high-risk women, including those with a history of previous GDM, advanced maternal age, family history of diabetes, pre-pregnancy weight gain and above the recommended gestational weight gain (GWG). The OGTT takes approximately 3 h and is considered expensive, unpleasant and time consuming [10]. Notably, there is an increasing number of undiagnosed cases of GDM in young pregnant women with potentially adverse consequences in mother and neonate [8,11].

Maternal obesity may also affect long-term offspring outcomes caused by foetal exposure to increased levels of glucose, insulin, lipids, and inflammatory cytokines during development [12]. This has been linked to permanent changes in foetal genetic programming, leading to obesity related chronic health conditions during childhood and as adults, including type 2 diabetes and cardiovascular disease [12]. Further evidence from long-term follow-up studies is required to determine the impact of maternal obesity on epigenetic changes to the offspring.

Local antenatal protocols identify pregnancies in women living with a body mass index (BMI) above 40 kg/m^2^ as high risk and women are transferred to specialist antenatal clinic facilities for the duration of their pregnancy. Transfer often means travelling large distances for clinic appointments and separation from families prior to birth.

A recent internal survey of midwives in the antenatal clinics in NW Tasmania, investigating barriers to weighing women and communicating the importance of monitoring weight gain during pregnancy. Results were mixed with some midwives feeling uncomfortable about weighing women due to the stigma around obesity, others concerned they may upset women, or uncertain of how to ‘start the conversation’. Some midwives stated that their personal struggle with weight affected their ability to communicate the importance of monitoring weight.

Given the range of complications linked to GDM and suboptimal weight gain during pregnancy including the potential long-term effects on the offspring, it is essential to develop strategies to optimise management, commencing with effective identification of those at high risk and providing women with adequate explanation of why it is important to manage GDM and monitor weight gain during pregnancy. Further, evidence on the impact of not being screened for GDM and the effects on pregnancy and foetal outcomes is limited. Services to address this deficit of screening need to be tailored to the requirements of rural remote regions of Australia. A service improvement project was initiated to determine how to proceed.

In 2018, antenatal and diabetes clinicians in NW Tasmania suggested that health service improvement was needed in three related areas: (1) the number of women entering pregnancy with overweight and obesity; (2) the number of women gaining weight above recommendations during pregnancy; and (3) the number of women not being screened for GDM. An audit of obstetric records for the previous two years was conducted with aims to determine whether service improvement was necessary, to provide a baseline assessment against which plans for change could be made, and to evaluate the impact of changes on the women and their babies.

## 2. Materials and Methods

Study design and setting: This was a cross-sectional retrospective audit of obstetric data undertaken in two antenatal clinics in regional Tasmania. All out-of-hospital care, including antenatal visits and postnatal care in the region are provided by two hospitals. Antenatal clinics included an obstetrician, a midwife, a lactation consultant, and registered nurses specialising in pregnancy and birth. The study was conducted within the north-west (NW) Tasmania antenatal service confines as defined by post code, and ethics approval was sought and obtained from the Tasmania Health Research Ethics Committee (H0017427).

**Participants:** All pregnant women who attended the antenatal clinics for a pregnancy-related visit between 1 July 2016 and 30 June 2018 were included in the audit. The sample size and time frame were considered adequate to allow for variability within the current population, changes in services and represent the general population. Both private and public obstetric patients were included. Women with pre-existing type 1 or type 2 diabetes were excluded from the analysis of those screened for GDM.

**Data measurements:** Antenatal and postnatal obstetric data were collected retrospectively from the patient’s obstetric digital medical record (Obstetrix). This records a standard minimum dataset of categorical data-fields (presence or absence of events or diagnoses), dates (visits and events, such as estimated and actual date of delivery), and continuous data-fields (e.g., weight, height, age). Pre-pregnancy BMI was based on the maternal height and weight recorded at the first antenatal visit (see Additional Materials for a detailed description): self-reported pre-pregnancy BMI was not reliably recorded.

GWG in individual women was assessed retrospectively using a calculator based on the Institute of Medicine (IOM) recommendations. The minimum data required for this calculator are the:

(1) Estimated date of delivery, from which the precise gestational age at each antenatal visit can be calculated.
(2) Height of the mother, to enable pre-pregnancy BMI calculation, which governs recommendations for GWG.
(3) Date of each antenatal visit.
(4) Weight of the mother at the first and subsequent visits.

The calculator, an algorithm written in Microsoft Excel, converts the maximum and minimum GWG recommendations for the four initial BMI categories (BMI < 18.5, BMI 18.5–25, BMI > 25–30, BMI > 30 kg/m^2^) to a continuously changing set of recommendations across the BMI range. This is based on the differences between the individual mother’s pre-pregnancy BMI, with those of the IOM [13] by process of non-linear (power equation) regression. The calculator estimates the weight at conception, often not known, from the weight measured at the first antenatal visit and gestational age of that visit, by a process of back-extrapolation based on the assumed daily proportional rise in weight during the pregnancy: the GWG recommendations are based on the assumed pre-pregnancy BMI. It is assumed the GWG at the final antenatal weight measurement will rise proportionately until delivery.

**Outcome measures: Categorical:** (1) GWG above or below as per IOM recommendations; (2) birth weight above 4000 g and below 2500 g; (3) maternal co-morbidities (hypertension, pre-eclampsia, GDM, mental and emotional health); (4) events of delivery (Caesarean section, instrumental delivery, genital trauma, post-partum haemorrhage(PPH); (5) neonatal co-morbidities (stillbirth, prematurity, admission to special care nursery or neonatal ICU; neonatal hypoglycaemia; respiratory distress and other impacts of delivery); and potential predictors of those outcomes.

**Outcome measures: Continuous:** (1) birth weight; and (2) gestational age at delivery.

**Predictor measures:** (1) BMI class; (2) GWG status as recommended, above or below recommendations; (3) GDM screening status (screened negative, screened positive, not screened); (4) whether premature (32–35 weeks, or <32 weeks); (5) maternal age; (6) socio-economic status in the lower tertile of local population range (Socio-Economic Index For Australia (SEIFA) derived from the Australian Bureau of Statistics 2016 census Statistical Area 1 (the smallest published area) of mother’s place of residence); (7) current smoking status; (8) hypertension and/or pre-eclampsia.

**Statistical analyses:** The associations between categorical outcomes were performed by estimating absolute percentage outcome occurrence (95% confidence intervals [CIs]; *p*-values) and incidence rate ratios (IRR; 95%CIs; *p*-values) using Poisson regression adjusted for each predictor measure listed above. These excluded those where outcome and predictor are the same for the regression model. Comparable analyses of continuous outcomes were performed by estimating group means and mean differences (Δ; 95%CIs; *p*-values) using general linear modelling adjusted for the Poisson regression model covariates. Full covariate coefficient estimates are shown in additional materials. All analyses were performed using Stata MP2 Version 16.1 (StataCorp, College Station, TX, USA).

## 3. Results

### 3.1. Obesity Prevalence

Between 1 July 2016 and 30 June 2018, there were 2244 births in NW Tasmania. The audit included 2132 records and 112 records were excluded as they did not include information to calculate BMI of the mother at conception. Women with a previous GDM pregnancy (*n* = 88), pre-existing type 1 (*n* = 12) or type 2 diabetes (*n* = 12) were included in 2132 records analyzed. Of the total number of women, 44.5% (949 of 2132) had a BMI < 25 kg/m^2^ and the remaining 55.5% (1183 of 2132) had a BMI > 25 kg/m^2^ (classified overweight or obese). A total 28.9% (614 of 2132) of mothers were living with obesity at conception (BMI > 30 kg/m^2^).

Most women (1735 of 2132) had weight and height measurements taken during antenatal visits that allowed estimation of GWG (at least two antenatal weight measurements). A total of 43.5% of mothers who were measured during antenatal visits (754 of 1735) had GWG within recommendations, 43.6% (757 of 1735) had GWG above recommendations, and 12.9% (224 of 1735) had GWG below recommendations.

### 3.2. Adverse Events Related to the Mother’s BMI at Conception and GWG Status at Delivery

(See Table 1, and Tables S4–S24), in Supplementary Materials for full regression models).

BMI and GWG categories were associated with adverse health outcomes in mothers (Table 1; Tables S10-S16 in Additional Materials) and their babies (Table 1; Tables S17–S24). The risk of stillborn babies was three times higher in women with a BMI <25 kg/m^2^ who did not have an OGTT. Eleven of 13 cases of stillbirth occurred in women who were not screened for GDM. This effect was greatly diminished when adjusted for premature delivery: IRR for stillbirth was 1.15 (95%CI 0.38–3.51; *p*-value 0.80) (see Table S12 in Supplementary Materials). Eleven of the 13 cases of stillbirth occurred before 25 weeks, before all routine GDM screening undertaken was completed.

PPH was similar in women with overweight or obesity (255 of 1183 (21.6%); IRR 1.09; 95%CI 0.92, 1.29; *p* = 0.34) to in women with a lower BMI (182 of 949; 19.2%). Preeclampsia and hypertension were more common in women with overweight or obesity (113 of 1183 [9.6%]); IRR 1.85; 95%CI 1.27, 2.68; *p* = 0.0012) than in women in the lower BMI range (41 of 949 [4.3%]).

Psychological conditions (anxiety disorders/depression) were more prevalent in women with a higher BMI (463 of 1183 [39.1%]); IRR 1.20; 95%CI 1.07, 1.35; *p* = 0.019) than in women in the lower BMI range (314 of 949 [33.1%]) (see Table 1). A significant proportion of the women (39.3%) were diagnosed with a mental health disorder before, during or after pregnancy. A pre-existing condition such as an eating disorder or schizophrenia was identified in 63 (3.0%) women, while 61 (2.9%) had post-natal depression without pre-existing mental health condition recorded in the obstetric electronic health record. Mental health conditions were more common in women with overweight or obesity (see Table 1).

The results suggest caesarean births were higher in women who were morbidly obese at conception (BMI > 35 kg/m^2^) than in women with a lower BMI. Instrumental or assisted birth rates were similar across all groups (78 of 1183 (6.6%); IRR 0.79; 95%CI 0.58, 1.06; *p* = 0.12) compared to women with a low or healthy BMI (83 of 949 [8.7%]).

The association between the baby’s birth weight and the mother’s BMI and her GWG during pregnancy above and below recommendations was estimated (see Table 2, and Tables S4–S9 in additional materials). BMI at conception and GWG were positively associated with a higher mean baby weight and macrosomia. Underweight was associated independently with low GWG, while BMI at conception was not associated with the neonate being underweight: prematurity had an overwhelming impact on low birth weight.

### 3.3. Gestational Diabetes Mellitus, Oral Glucose Tolerance Testing, and Pregnancy Complications

An OGTT was completed by 1368 of 2132 mothers (64.2%) during their pregnancy. Unless otherwise recommended, GDM screening occurred around 24–26 weeks gestation. Therefore, screening was low in extremely premature babies: 28 of 37 mothers (75.7%) who delivered before 32 weeks were not screened for GDM, compared to 736 of 2095 of mothers (24.3%) who delivered after 32 weeks (IRR 2.17; 95%CI 1.80, 2.63; p < 0.0001). Of the 1368 mothers screened, 212 (15.5%) were diagnosed with GDM. There were 23 women with a history of type 1 (12) or type 2 (11) diabetes who did not require an OGTT due to preexisting diabetes. A total of 69.3% of mothers with GDM (127 of 212) had a BMI > 30 kg/m^2^. The rate of diagnosis of GDM was lowest in mothers with healthy or underweight BMIs, and the rate of GDM increased progressively across the BMI range (see Table 3).

Table 4 shows the effect of BMI class where BMI at conception could be calculated. Mothers with BMI > 22.5 kg/m^2^ were more likely to be screened for GDM (IRR 1.13; 95%CI 1.03, 1.23; *p* = 0.0076), especially those with BMI > 35 kg/m^2^ (IRR 1.24; 95%CI 1.11, 1.39; *p* = 0.0001), than mothers with BMI < 22.5 kg/m^2^, although this effect was not large.

A number of women (15.9%) with GDM had GWG below recommendations (IRR 1.73; 95%CI 1.25, 2.39; *p* = 0.0009) compared to 9.2% of women without GDM: women with GDM and obesity were actively encouraged to limit GWG as part of their GDM control therapy. Those women were also less likely to have GWG above recommendations (IRR 0.70; 95%CI 0.57, 0.87; *p* = 0.0010) for the same reason (see Tables S6 and S7 in Additional Materials). They delivered marginally earlier on average (Mean Δ −0.80 weeks; 95%CI −1.14, −0.46; *p* < 0.0001), and were more likely to be delivered by Caesarean section (IRR 1.32; 95%CI 1.08, 1.42; *p* = 0.0018), again consistent with active management protocols for GDM. Babies of mothers with GDM were more likely to suffer episodes of neonatal hypoglycaemia (IRR 5.93; 95%CI 3.39, 10.4; *p* < 0.0001), and more likely to be admitted to the Special Care Nursery (IRR 1.76; 95%CI 1.30, 2.38; *p* = 0.0003) either to manage or to monitor for neonatal hypoglycaemia.

### 3.4. Miscellaneous Associations

**Macrosomia and microsomia:** Ninety women in this audit had a baseline BMI > 40 kg/m^2^ (morbid obesity). Of this group, 73% (*n* = 66) gained weight during pregnancy and 16 (24%) were diagnosed with GDM. The remaining 22 women (24%) maintained the same weight or lost weight, of this group and 11 (50%) were diagnosed with GDM.

Of the women with morbid obesity diagnosed with GDM (*n* = 27), 18 delivered a baby of average weight (2499–3999 g), six with macrosomia (>4000 g), and three with small for gestational weight (SGW) (<2499 g). Of note, for the entire group of women living with morbid obesity who gained weight during pregnancy (*n* = 66), 43 (65%) delivered babies with average weight, 11 (17%) delivered babies with macrosomia, and 12 delivered babies (18%) weighing <2499 g. In contrast, for women with morbid obesity who maintained their pre-pregnancy weight or lost weight (*n* = 21), 17 (77.2%) delivered babies with average weight, 4 (18.1%) delivered babies with macrosomia, and none delivered babies <2499 g.

In babies born after 32 weeks, delivery weight, weight above 4000 g and weight below 2500 g, showed similar associations with the mother’s BMI at conception and GWG gain above and below recommendations (see Table 5). However, excessive GWG was associated with a reduced risk of delivering SGW babies <2500 g (2.3%; IRR 0.60; 95%CI 0.41, 0.87; *p* = 0.0068), and GWG below recommendations was associated with an increased risk of SGW <2500 g babies (5.9%; IRR 1.54; 95%CI 1.00, 2.36; *p* = 0.050) compared to GWG within recommendations (3.9%) (see Table S6 Additional materials).

## 4. Discussion

Our findings indicate that over 50% of pregnant women who presented to antenatal clinic were overweight and almost 30% obese. Both values are consistent with national rates and a similar audit of pregnant women presenting in rural Queensland [14].

This audit confirmed an increased risk of adverse health outcomes for women and their babies as maternal BMI increased, including diagnosis of GDM, higher birthweight babies (≥4000 g) and pre-eclampsia. Further, in the present cohort, 35% of women (*n* = 747) did not have an OGTT performed during pregnancy, with the largest proportion of these women falling in the lower BMI range. Recent findings from a study in rural Western Australia reported that only half of all pregnant women had an OGTT; the reasons for not having the test being unclear [15].

The difference in proportion of women who did not have an OGTT was highest in women with a lower BMI suggesting a perception that women with a lower BMI may have a reduced risk of GDM. This perception explained only a minority of the screening deficit. Quaresima et al. [16] reported that pregnant women had a poor knowledge regarding the effects of GDM on mothers and neonates and recommended that in high-risk pregnancies, early detection of GDM between 6–18 weeks, is crucial to prevent LGA babies. Furthermore, undiagnosed GDM may also pose an increased risk of babies born with hypoglycaemia, subsequent admission to NICU (5.8%) and a threefold increase in stillbirth, particularly in those women with a BMI <25 kg/m^2^ [2,17] although the confounding effect of premature delivery before planned OGTT may explain some of this effect. In rural and regional areas, the potential difficulties associated with the test, including time, travel distance, and family commitments, along with expected side effects such as nausea, may affect women’s uptake of GDM screening.

Nine percent of women in our audit were diagnosed with GDM, lower than the current national figure of 12–14% [18]. However, given the low participation rates in OGTT screening, this may not be an accurate indication of the GDM prevalence.

Attention to screening processes requires urgent attention to ensure women are screened for GDM as required by the national standards or to develop alternative screening pathways for women unable to or not agreeing to have an OGTT, to improve the rate of diagnosis [19]. A revision for the local protocols for GDM screening was undertaken, which will be evaluated in a future audit.

Current clinical care guidelines indicate that, women should ideally be counselled before conception about the increased risks associated with obesity in pregnancy and be encouraged to make lifestyle changes where necessary [6,20]. Providing additional support for all women to improve their lifestyle through dietary interventions and increasing physical activity, may have a substantial impact on reducing adverse outcomes for mothers and neonate [20]. In NW Tasmania, the relative poverty, lower educational attainment, and shortage of health professionals may limit the effectiveness of the standard approach to such service delivery, requiring modifications for the local population. The higher rate of mental health conditions amongst women with higher BMI will add to those counselling skills requirements and may require psychological therapies and medications to meet the women’s emotional and mental health needs.

Data from our audit are consistent with the American College of Obstetricians and Gynecologists’ (ACOG) [21] assertion that the IOM GWG targets for pregnant women with obesity were too high [22]. The data in our study indicate that women who remained weight-neutral, or those who lost weight, did not deliver SGA babies. However, women with a lower BMI (<25 kg/m^2^) with poor GWG were at greater risk of having an SGA baby, and three-fold greater likelihood of having a stillborn baby (*n* = 7) compared to the higher BMI categories.

Individualized care and sound clinical judgment are required to manage a woman with overweight or obesity who are gaining less weight than recommended but has an appropriately growing fetus [6,20,22]. It is also important to highlight that not all pregnant women with obesity have adverse pregnancy outcomes [20]. Gaining less than recommended weight according to national and international guidelines should be assessed and may be due to several factors, including: (i) improvement in food choices, choosing nutrient-dense healthier options and fewer high-calorie nutrient-deficient foods and drinks; (ii) increased physical activity; (iii) severe hyperemesis; and (iv) excessive dietary restrictions and activity [21]. Some of these may be good and some bad for the mother and/or baby.

Assessment and care options will depend on the clinical circumstances, the presence of comorbidities, and the resources and services available at each healthcare facility. Roman et al. [17] concluded that obesity is more important than GWG in the development of pre-eclampsia and GDM and refer to the importance of pre-conception weight reduction and counselling, in reducing morbidity in pregnancy. However, a meta-analysis of the association between GWG and adverse maternal and neonatal outcomes, was related to the risk of complications in pregnancy, weight retention and obesity in the offspring [23].

A limitation of this study was the self-reported pre-pregnancy weight and its consequences for calculating BMI. A method for back-extrapolation of weight at conception was used as part of an electronic GWG calculator developed for this project (see Additional Materials); however, this methodology has yet to be validated. Anthropometric measures performed under routine clinical conditions are potential sources of bias. The study’s retrospective nature and the reliance on routine clinical records are further possible limitation and strength of this study, being both less rigorous and more clinically relevant. Unfortunately, the effect of parity on maternal weight was not extracted from the electronic database, which limited our understanding of the effect of repeated pregnancies with excess weight gain, as opposed to excess weight gain between pregnancies. A longitudinal study of women when pregnant and when not pregnant would be required to understand these issues.

This audit highlights a significant gap: a failure to screen all pregnant women for GDM using an OGTT in regional rural Tasmania. Further investigation is urgently required to understand to improve uptake of this test. Furthermore, it is vital that future studies examine pre-pregnancy weight status, GWG patterns, maternal fetal adverse outcomes, postnatal prevalence of obesity in infants, and other conditions pre-disposing to epigenetic changes in infants and early childhood as a result of suboptimal GWG.

A discrepancy exists between health care providers (HCP) and reported communication with women about GWG [23]. Midwives reported lack of time, weight stigma and lack of confidence to engage in the conversation about weight gain, as barriers to providing advice about weight gain during pregnancy, particularly to women who are overweight or living with obesity. Conversely, women reported they did not receive counselling about GWG [23]. This further highlights the need for HCP to improve their skills, confidence, and knowledge about the importance of monitoring gestational weight gain and promoting healthy lifestyles during pregnancy.

## 5. Conclusions

There is a high prevalence of overweight and obesity among pregnant women in rural regional Tasmania. Supportive, and patient-centered discussion regarding the importance of GWG should be introduced into antenatal clinics. Midwives are particularly well placed to offer this support. Future upskilling of midwives to provide healthy lifestyle advice may assist in reducing the risk of adverse health outcomes during pregnancy and the increased risk of obesity and metabolic disease in their offspring into the future. The higher rate of mental health conditions amongst women with higher BMI will add to those skills requirements. The lower rate of screening for GDM is a concern, and actions need to be taken to address this deficiency.

## Figures and Tables

**Table 1:** Maternal associations of different BMI and GWG categories.

Predictors		Cases	Total	Case % ^3^	IRR ^3^	95%CI	*p*-Value
Outcome: hypertension of pregnancy or pre-eclampsia							
Mother’s BMI at conception ^1^	<18.5	2	64	4.1	0.99	(0.22, 4.42)	0.99
	18.5–25	31	704	4.1	1.00		
	25–30	30	469	5.2	1.26	(0.75, 2.12)	0.38
	30–35	18	244	5.5	1.34	(0.75, 2.38)	0.32
	>35	36	254	10.8	2.62	(1.58, 4.35)	0.0002
GWG recommendations ^2^	Within	37	754	4.1	1.00		
	Above	70	757	8.6	2.07	(1.39, 3.10)	0.0004
	Below	10	224	2.9	0.69	(0.35, 1.37)	0.29
Outcome: anxiety, depression, and/or postnatal depression							
Mother’s BMI at conception ^1^	<18.5	31	64	39.4	1.32	(1.02, 1.70)	0.033
	18.5–25	227	704	29.9	1.00		
	25–30	162	469	32.9	1.10	(0.94, 1.29)	0.24
	30–35	107	244	41.5	1.39	(1.17, 1.66)	0.0003
	>35	122	254	45.2	1.51	(1.27, 1.79)	<0.0001
GWG recommendations ^2^	Within	268	754	29.9	1.00		
	Above	278	757	31.2	1.05	(0.92, 1.19)	0.51
	Below	103	224	33.8	1.13	(0.96, 1.34)	0.15
Outcome: Caesarean births							
Mother’s BMI at conception ^1^	<18.5	13	64	16.9	0.64	(0.39, 1.05)	0.080
	18.5–25	243	704	26.5	1.00		
	25–30	175	469	27.7	1.05	(0.90, 1.22)	0.57
	30–35	88	244	26.9	1.02	(0.84, 1.23)	0.86
	>35	121	254	33.4	1.26	(1.07, 1.49)	0.0065
GWG recommendations ^2^	Within	252	754	26.5	1.00		
	Above	313	757	32.8	1.24	(1.08, 1.42)	0.0017
	Below	75	224	25.9	0.98	(0.79, 1.21)	0.84

^1.^ A total of 1735 mothers had adequate measurements to estimate her BMI at conception. Percentage of mothers in each BMI range: <18.5, 3.7%; 18.5–25, 40.6%; 25–30, 27.0%; 30–35, 14.1%; 35+, 14.6%. ^2.^ GWG categories: based on IOM (renamed National Academy of Medicine) GWG recommendations, 2009. ^3.^ Absolute rate and incidence rate ratio (IRR; 95% confidence interval; *p*-values) estimated using Poisson regression adjusted for age of mother, whether screened for GDM and result, prematurity before and after 32 weeks, low SEIFA, smoking, and hypertension/pre-eclampsia. The healthy BMI range (18.5–25 kg/m^2^) was used as the comparator for the mean IRR comparisons. (Full model results are shown in Tables S4.3–S4.5 in Supplementary Materials).

**Table 2:** Associations between measures of the baby’s birth weight and its mother’s pre-pregnancy BMI and GWG by the time of delivery.

Predictor ^1^		*n*	Mean Weight (g) ^4^	SD	Mean Δ (g) ^4^	95%CI	*p*-Value
Outcome: baby birth weight							
Mother’s BMI at conception ^2^	<18.5	64	3228	720	−145	(−260, −30)	0.013
	18.5–25	704	3373	657	0		
	25–30	469	3471	659	98	(39, 156)	0.0011
	30–35	244	3454	674	81	(6, 156)	0.033
	35+	254	3546	656	173	(95, 251)	<0.0001
GWG recommendations ^3^	Within	754	3373	728	0		
	Above	757	3541	569	167	(116, 219)	<0.0001
	Below	224	3238	675	−135	(−213, −58)	<0.0001
**Predictor ^1^**		***n***	***N***	**% in Group with Condition ^5^**	**IRR ^5^**	**95%CI**	***p*-Value**
Outcome: macrosomia (birth weight > 4000 g)							
Mother’s BMI at conception ^2^	<18.5	0	64	0.0%	0.00	(0.00, 0.00)	<0.0001
	18.5–25	77	704	9.0%	1.00		
	25–30	89	469	14.3%	1.59	(1.20, 2.10)	0.0011
	30–35	37	244	12.0%	1.33	(0.92, 1.92)	0.12
	35+	47	254	16.0%	1.77	(1.26, 2.49)	0.0010
GWG recommendations ^3^	Within	82	754	9.0%	1.00		
	Above	149	757	14.9%	1.66	(1.30, 2.12)	<0.0001
	Below	19	224	6.4%	0.71	(0.44, 1.13)	0.15
Outcome: microsomia (birth weight < 2500 g)							
Mother’s BMI at conception ^2^	<18.5	6	61	3.4%	0.73	(0.41, 1.32)	0.30
	18.5–25	49	691	4.7%	1.00		
	25–30	27	462	5.1%	1.08	(0.73, 1.61)	0.71
	30–35	13	239	5.7%	1.21	(0.69, 2.11)	0.52
	35+	12	251	4.0%	0.85	(0.47, 1.53)	0.59
GWG recommendations ^3^	Within	54	729	4.7%	1.00		
	Above	31	755	2.8%	0.59	(0.41, 0.86)	0.0060
	Below	22	220	7.1%	1.50	(0.98, 2.30)	0.061

^1.^ Predictors included in each model: corrected BMI at conception; actual delivery GWG against recommendations; GDM screening status; prematurity; age of mother; low SEIFA tertile; smoking; any hypertension/PET. ^2.^ A total of 1735 mothers had adequate measurements to estimate their pre-pregnancy BMI. Mother’s BMI at the time of conception, and the rate of GWG. ^3.^ GWG categories: based on IOM (renamed National Academy of Medicine) GWG recommendations, 2009. ^4.^ The mean birth weights of the babies of women in each BMI range were estimated using general linear modelling, shown as the mean (SD) and mean difference (Δ; 95% confidence intervals; *p*-values) compared to a healthy weight and GWG within recommendations, adjusted for predictors^5^ included in each model. ^5.^ The rate and relative rate of the outcome conditions (macrosomia and microsomia) were estimated using Poisson regression, shown as percentage (95%CI) and incidence rate ratio (IRR; 95% confidence intervals; *p*-values), adjusted of predictors^1^ included in each model.

**Table 3:** Numbers (%) and relative risk (IRR) of mothers screened for GDM in different BMI ranges.

BMI Class	BMI Range (kg/m^2^)	Screened	Total	%	IRR ^1^	95%CI	*p*-Value
Underweight	<18.5	41	75	54.7	1.00	(0.80, 1.26)	0.98
Healthy	18.5–22.5	266	487	54.6	1.00		
	22.5–25	237	387	61.2	1.13	(1.01, 1.28)	0.036
Overweight	25–30	348	569	61.2	1.08	(0.97, 1.21)	0.16
Obese Class 1	30–35	195	316	61.7	1.15	(1.01, 1.30)	0.029
Obese Class 2	35–40	122	173	70.5	1.25	(1.09, 1.43)	0.0013
Morbid obesity	40–45	50	77	64.9	1.20	(1.00, 1.43)	0.054
	45+	29	48	60.4	1.16	(0.91, 1.47)	0.23

Likelihood of undergoing GDM screening in other BMI groups was compared to mothers in the BMI range 18.5–22.5 kg/m^2^; incidence rate ratio (^1.^ IRR; 95% confidence intervals; *p*-values) was estimated using Poisson regression, adjusted for maternal age.

**Table 4:** Diagnosis of GDM in different BMI ranges and GWG categories in mothers screened for GDM during current pregnancy.

BMI Range (kg/m^2^)		GDM Cases	Total in BMI Range	GDM Rate ^3^	IRR ^3^	95%CI	*p*-Value
Mother’s BMI at conception ^1^	<18.5	1	37	2.8%	0.56	(0.07, 4.28)	0.58
	18.5–25	20	429	5.0%	1.00		
	25–30	29	292	11.2%	2.22	(1.28, 3.83)	0.0044
	30–35	22	161	14.0%	2.78	(1.56, 4.93)	<0.0001
	35–40	24	102	22.6%	4.47	(2.60, 7.70)	<0.0001
	40–45	18	48	35.3%	7.00	(3.94, 12.5)	<0.0001
	45+	10	27	33.1%	6.56	(3.29, 13.1)	<0.0001
GWG recommendations ^2^	Within	44	416	5.0%	1.00		
	Above	45	532	3.6%	0.72	(0.48, 1.06)	0.098
	Below	35	148	7.6%	1.50	(1.00, 2.25)	0.048

^1.^ A total of 1096 mothers had both GDM screening and adequate weight/height measurements to estimate her BMI at conception: the percentage of women with GDM in each BMI category was <18.5, 3.5%; 18.5–25, 40.5%; 25–30, 27.6%; 30–35, 15.2%; 35–40, 9.6%; 40–45, 4.5%; 45+, 2.5%. The table shows the number of women with GDM plus those with Type 1 and Type 2 diabetes mellitus; the total number of mothers; and percentage of mothers with GDM in each category. ^2.^ GWG categories: based on IOM (renamed National Academy of Medicine) GWG recommendations, 2009. ^3.^ The incidence rate ratio (IRR; 95% confidence intervals; *p*-values) of women in each BMI range diagnosed with GDM was estimated using Poisson regression adjusted for prematurity; age of mother; low SEIFA; current smoking; hypertension and/or pre-eclampsia; shown as both percentage of mothers in each BMI range (95% confidence intervals).

**Table 5:** Neonatal complication associated with from BMI and GWG categories.

Predictors		Cases	Total	Case % ^3^	IRR ^3^	95%CI	*p*-Value
Outcome: macrosomia							
Mother’s BMI at conception ^1^	<18.5	0	64	0.0%	0.00	(0.00, 0.00)	<0.0001
	18.5–25	77	704	9.0%	1.00		
	25–30	89	469	14.3%	1.59	(1.20, 2.10)	0.0011
	30–35	37	244	12.0%	1.33	(0.92, 1.92)	0.12
	>35	47	254	16.0%	1.77	(1.26, 2.49)	0.0010
	Within	82	754	9.0%	1.00		
GWG recommendations ^2^	Above	149	757	14.9%	1.66	(1.30, 2.12)	<0.0001
	Below	19	224	6.4%	0.71	(0.44, 1.13)	0.15
Outcome: neonatal hypoglycaemia							
Mother’s BMI at conception ^1^	<18.5	0	64	0.00%	0.00	(0.00, 0.00)	<0.0001
	18.5–25	19	704	1.42%	1.00		
	25–30	23	469	2.29%	1.61	(0.89, 2.91)	0.12
	30–35	14	244	2.38%	1.67	(0.81, 3.44)	0.17
	>35	19	254	2.14%	1.50	(0.80, 2.82)	0.21
GWG recommendations ^2^	Within	31	754	1.42%	1.00		
	Above	28	757	1.34%	0.94	(0.56, 1.59)	0.82
	Below	16	224	2.16%	1.52	(0.82, 2.80)	0.19
Outcome: neonatal respiratory distress							
Mother’s BMI at conception ^1^	<18.5	4	64	3.5%	0.87	(0.36, 2.10)	0.76
	18.5–25	36	704	4.1%	1.00		
	25–30	28	469	5.2%	1.29	(0.81, 2.05)	0.29
	30–35	12	244	4.2%	1.03	(0.55, 1.93)	0.92
	>35	16	254	4.8%	1.19	(0.67, 2.11)	0.56
GWG recommendations ^2^	Within	39	754	4.1%	1.00		
	Above	36	757	4.4%	1.08	(0.69, 1.70)	0.74
	Below	21	224	8.6%	2.12	(1.30, 3.44)	0.0024
Outcome: admission to the Neonatal Intensive Care Unit							
Mother’s BMI at conception ^1^	<18.5	4	64	0.6%	0.92	(0.41, 2.03)	0.83
	18.5–25	24	704	0.7%	1.00		
	25–30	10	469	0.5%	0.75	(0.40, 1.40)	0.36
	30–35	7	244	0.8%	1.10	(0.46, 2.64)	0.84
	>35	12	254	1.1%	1.54	(0.92, 2.58)	0.097
	Within	27	754	0.7%	1.00		
GWG recommendations ^2^	Above	19	757	0.8%	1.18	(0.70, 1.99)	0.53
	Below	11	224	1.5%	2.05	(1.24, 3.41)	0.0053

^1^ A total of 1735 mothers had adequate measurements to estimate her BMI at conception. Percentage of mothers in each BMI range: <18.5, 3.7%; 18.5–25, 40.6%; 25–30, 27.0%; 30–35, 14.1%; 35+, 14.6%. ^2^ GWG categories: based on Institute of Medicine (renamed National Academy of Medicine) GWG recommendations, 2009. ^3^ Absolute rate and incidence rate ratio (IRR; 95% confidence interval; *p*-values) estimated using Poisson regression adjusted for age of mother, whether screened for GDM and result, prematurity before and after 32 weeks, low SEIFA, smoking, and hypertension/pre-eclampsia. The healthy BMI range (18.5–25 kg/m^2^) was used as the comparator for the mean IRR comparisons.

## Data Availability

Access to data will only be permitted by approved research investigators as advised by ethics approval. There are no publicly archived data sets generated during this study.

## Supplementary Materials

The following are available online at https://www.mdpi.com/article/10.3390/ijerph182212006/s1, Table S1: Standard Classifications and recommendations; Table S2: Birth weight classifications; Table S3: Recommended guidelines for weight gain during pregnancy; Table S4: The effect of BMI status at conception and GWG at delivery on the birth weight of the baby; Table S5: The effect of BMI status at conception and GWG at delivery on the gestational age at delivery; Table S6: The effect of BMI status at conception on the rate of gestational weight gain below recommendations; Table S7: The effect of BMI status at conception on the rate of gestational weight gain above recommendations; Table S8: The effect of BMI status at conception and GWG at delivery on the rate of macrosomia; Table S9: The effect of BMI status at conception and GWG at delivery on the rate of birth weight less than 2500 g; Table S10: The effect of BMI status at conception and GWG at delivery on the rate of gestational diabetes mellitus in this pregnancy or prior diabetes mellitus; Table S11: The effect of BMI status at conception and GWG at delivery on the rate of maternal hypertension and/or pre-eclampsia during; Table S12: The effect of BMI status at conception and GWG at delivery on the rate of maternal mental health disorders during pregnancy and delivery period; Table S13: The effect of BMI status at conception and GWG at delivery on the rate of delivery by Caesarean section; Table S14: The effect of BMI status at conception and GWG at delivery on the rate of instrumental delivery; Table S15: The effect of BMI status at conception and GWG at delivery on the rate of Moderate-to-severe genital trauma during delivery; Table S16: The effect of BMI status at conception and GWG at delivery on the rate of post-partum haemorrhage; Table S17: The effect of BMI status at conception and GWG at delivery on the rate of stillbirth; Table S18: The effect of BMI status at conception and GWG at delivery on the rate of prematurity before 32 weeks; Table S19: The effect of BMI status at conception and GWG at delivery on the rate of immediate admission to the Special Care Nursery; Table S20: The effect of BMI status at conception and GWG at delivery on the rate of immediate admission to the Neonatal Intensive Care Unit; Table S21: The effect of BMI status at conception and GWG at delivery on the rate of neonatal hypoglycaemia; Table S22: The effect of BMI status at conception and GWG at delivery on the rate of neonatal respiratory distress; Table S23: The effect of BMI status at conception and GWG at delivery on the rate of other neonatal complications; Table S24: The effect of BMI status at conception and GWG at delivery on the rate of other birth injuries to baby.
