# Supplementary material for: An Audit of Pre-Pregnancy Maternal Obesity and Diabetes Screening in Rural Regional Tasmania and Its Impact on Pregnancy and Neonatal Outcomes
Source: Int J Environ Res Public Health. 2021 Nov 16;18(22):12006. doi: 10.3390/ijerph182212006 (PMC8619569; doi:10.3390/ijerph182212006)
Supplement: Supplementary file 1 [file ijerph-18-12006-s001.zip › ijerph-1412780-supplementary.pdf]

Additional Materials.

Standard classifications and recommendations

Table S1: Diagnostic criteria for GDM

**OGTT**	
Fasting glucose	≥5.1 mmol/L
1-hr glucose	≥10 mmol/L
2-hr glucose	≥8.5 mmol/L
**HbA1c**	**≥5.9%** (1st trimester)
Fasting BG	≥ **5.1** (post 12 weeks)

GDM was categorised according to the current recommendations for screening of GDM in Australia, a 3-hour OGTT recommended at 24–28 weeks of pregnancy (see Table 1)[16] and alternative HbA1c and FBG.

Table S2: Birth weight classifications

**Human birth weight classifications**											
**Birth weight** (grams)	less 500	500 - 999	1,000 - 1,499	1,500 - 1,999	2,000 - 2,499	2,500 - 2,999	3,000 - 3,499	3,500 - 3,999	4,000 - 4,499	4,500 - 4,999	>5,000
**Classification (birth weight)**	Extremely low		Very low	Low		Normal			High		

Birth weight was classified according to published Human Birth Weight Classifications (see Table 2)[17].

Table S3: Recommended guidelines for weight gain during pregnancy

**BMI**	**Classification**	**Recommended Gestational Weight Gain**
**Less than**	Underweight	12.5 – 18.0 kg
**18.5-24.9**	Normal	11.5 – 16.0 kg
**25-29.9**	Overweight	6.8 – 11.3 kg
**30 to 34.99**	Obesity class 1	5.0 – 9.0 kg
**35 to 39.99**	Obesity class 2	
**More than 40**	Morbidly Obese	

Recommendations of the Royal Australian and New Zealand College of Obstetricians and Gynaecologists 2009

Classification of BMI according to World Health Organisation (WHO)

Supplementary Results Tables including full regression model covariates

Footnotes to Supplementary Tables S4and S5

[1] BMI at conception, and gestational weight gain (GWG) status at delivery, were estimated using the GWG calculator. Mothers were categorised as having GWG values within, above or below recommendations the Institute of Medicine Guidelines at the time of delivery.

[2] 1,368 mothers were screened for gestational diabetes mellitus (GDM) during pregnancy or had been diagnosed with diabetes (Type 1 or Type 2) prior to this pregnancy. 714 mothers were not screened for GDM during this pregnancy

[3] Socio-economic Index for Australia (SEIFA) for disadvantage (Australian Bureau of Statistics 2016 census) was estimated using the Statistical Area 1 (ABS census area definition) place of residence of the mother during pregnancy. The Low SEIFA group had scores less than 898, a third: two-thirds partition

[4] 2,132 mothers had adequate weight/height measurements to estimate her BMI at conception using the GWG calculator (at least one height and weight measurement: Tables show the number (Total) and % of mothers in each BMI range; and number of women with GDM plus those with Type1 and Type2 diabetes mellitus (GDM).

[5] The mean birth weight of the babies of women in each BMI range (and other predictors) were estimated using general linear modelling adjusted for maternal age range; low SEIFA; current smoking; GDM screening status; prematurity; hypertension and/or pre-eclampsia. The results shown were mean (standard deviation SD) and mean difference (Δ; 95% confidence intervals; P-values). The predictor grouping used as the comparator for each covariate for the comparisons was the one with a Δ of 0.00. These full model results correspond with the summary results shown in Table 4 in the main paper.

Footnotes to Supplementary Tables S6 to S22

[1] BMI at conception, and gestational weight gain (GWG) status at delivery, were estimated using the GWG calculator. Mothers were categorised as having GWG values within, above or below recommendations the Institute of Medicine Guidelines at the time of delivery.

[2] 1,368 mothers were screened for gestational diabetes mellitus (GDM) during pregnancy or had been diagnosed with diabetes (Type 1 or Type 2) prior to this pregnancy. 714 mothers were not screened for GDM during this pregnancy

[3] Socio-economic Index for Australia (SEIFA) for disadvantage (Australian Bureau of Statistics 2016 census) was estimated using the Statistical Area 1 (ABS census area definition) place of residence of the mother during pregnancy. The Low SEIFA group had scores less than 898, a third: two-thirds partition

[4] 2,132 mothers had adequate weight/height measurements to estimate her BMI at conception using the GWG calculator: Tables show the number (Total) and % of mothers in each BMI range; and number of women with GDM plus those with Type1 and Type2 diabetes mellitus (GDM).

[5] The percentage of women diagnosed with the outcome condition in each predictor range (BMI and GWG ranges) in whom the outcome condition occurred was estimated using Poisson regression adjusted for the covariates shown in the models; shown as both percentage of mothers with the condition of each analysis (95% confidence intervals), and incidence rate ratio (IRR; 95%CI; P-values). The healthy BMI range (18.5-25 $kg.m^2$) was used as the comparator for the mean IRR comparisons. These full model results correspond with the summary results shown in Tables 4, 5 and 6 in the main paper.

Tables S4 to S9 show the associations between baby weight and duration of pregnancy as outcomes and maternal predictors of those outcomes. These are expansions of Table 1 in the main paper

Table S4: The effect of BMI status at conception and GWG at delivery on the birth weight of baby

Predictor	Predictor groupings	Group % BMI [4]	n	Mean weight (kg) [5]	SD	Mean Δ (kg) [5]	95%CI	P-value
		Outcome: Baby birth weight						
BMI (kg.m²) at conception [1]	<18.5	3.7%	64	3228	720	-145	(-260, -30)	0.013
	18.5-25	40.6%	704	3373	657	0		
	25-30	27.0%	469	3471	659	98	(39, 156)	0.0011
	30-35	14.1%	244	3454	674	81	(6, 156)	0.033
	35+	14.6%	254	3546	656	173	(95, 251)	<0.0001
Birth GWG versus recommendations [1]	Within	43.5%	754	3373	728	0		
	Above	43.6%	757	3541	569	167	(116, 219)	<0.0001
	Below	12.9%	224	3238	675	-135	(-213, -58)	<0.0001
DM screening status & result [2]	GDM excluded	56.0%	972	3373	592	0		
	GDM diagnosed	10.5%	183	3436	665	63	(-27, 153)	0.17
	GDM not screened	33.4%	580	3320	773	-53	(-105, -2)	0.043
Whether premature	No prematurity	95.2%	1,651	3373	532	0		
	Prem: 32-35 weeks	3.1%	53	2078	485	-1295	(-1412, -1178)	<0.0001
	Prem: <32 weeks	1.8%	31	923	487	-2450	(-2627, -2272)	<0.0001
Age of mother (years)	Mother <25	28.2%	490	3373	688	0		
	Mother 25-35	59.7%	1,035	3404	643	31	(-24, 86)	0.27
	Mother 35+	12.1%	210	3436	746	63	(-21, 146)	0.14
SEIFA band [3]	High/middle SEIFA	65.2%	1,132	3373	634	0		
	Low SEIFA	34.8%	603	3357	732	-17	(-69, 36)	0.54
Smoking	Current non-smoker	74.3%	1,289	3373	617	0		
	Current smoker	25.7%	446	3287	762	-86	(-111, -62)	<0.0001
Hypertension and/or Pre-eclampsia	No HT/P-Ecl	93.3%	1,618	3373	672	0		
	HT/P-Ecl	6.7%	117	3147	638	-226	(-324, -129)	<0.0001

Table S5: The effect of BMI status at conception and GWG at delivery on the gestational age at delivery

Predictor	Predictor groupings	Group % BMI [4]	n	Mean age (weeks) [5]	SD	Mean Δ (weeks) [5]	95%CI	P-value
			Outcome: Gestational age at delivery					
BMI (kg.m^2) at conception [1]	<18.5	3.7%	64	38.3	3.5	-0.73	(-1.60, 0.13)	0.097
	18.5-25	40.6%	704	39.0	2.4	0.00		
	25-30	27.0%	469	39.1	2.4	0.09	(-0.18, 0.36)	0.52
	30-35	14.1%	244	39.0	2.7	0.01	(-0.37, 0.40)	0.95
	35+	14.6%	254	39.0	2.1	-0.02	(-0.37, 0.33)	0.89
Birth GWG versus recommendations [1]	Within	43.5%	754	39.0	3.0	0.00		
	Above	43.6%	757	39.6	1.8	0.60	(0.37, 0.84)	<0.0001
	Below	12.9%	224	39.3	2.4	0.33	(-0.05, 0.71)	0.090
DM screening status & result [2]	GDM excluded	56.0%	972	39.0	1.8	0.00		
	GDM diagnosed	10.5%	183	38.2	2.0	-0.80	(-1.14, -0.46)	<0.0001
	GDM not screened	33.4%	580	38.4	3.3	-0.64	(-0.92, -0.36)	<0.0001
Age of mother (years)	Mother <25	28.2%	490	39.0	2.5	0.00		
	Mother 25-35	59.7%	1,035	39.0	2.3	0.03	(-0.24, 0.29)	0.85
	Mother 35+	12.1%	210	38.6	3.0	-0.37	(-0.84, 0.10)	0.12
SEIFA band [3]	High/middle SEIFA	65.2%	1,132	39.0	2.3	0.00		
	Low SEIFA	34.8%	603	38.9	2.8	-0.12	(-0.38, 0.13)	0.34
Smoking	Current non-smoker	74.3%	1,289	39.0	2.2	0.00		
	Current smoker	25.7%	446	38.7	3.1	-0.31	(-0.44, -0.17)	<0.0001
Hypertension and/or Pre-eclampsia	No HT/P-Ecl	93.3%	1,618	39.0	2.5	0.00		
	HT/P-Ecl	6.7%	117	38.3	1.8	-0.73	(-1.07, -0.38)	<0.0001

Table S6: The effect of BMI status at conception on the rate of gestational weight gain below recommendations

Predictor	Predictor groupings	Group % BMI [4]	n	N	% in group with condition [5]	95%CI	IRR [5]	95%CI	P-value
Outcome: Gestational weight gain below recommendations									
BMI (kg.m^2) at conception [1]	<18.5	3.7%	15	64	21.2%	(13.0%, 34.6%)	2.31	(1.42, 3.76)	0.0008
	18.5-25	40.6%	71	704	9.2%	(6.7%, 12.7%)	1.00		
	25-30	27.0%	41	469	7.8%	(5.4%, 11.3%)	0.85	(0.58, 1.22)	0.37
	30-35	14.1%	37	244	13.2%	(9.1%, 19.1%)	1.43	(0.99, 2.07)	0.056
	35+	14.6%	60	254	18.7%	(13.4%, 26.2%)	2.03	(1.45, 2.84)	<0.0001
DM screening status & result [2]	GDM excluded	56.0%	113	972	9.2%	(6.7%, 12.7%)	1.00		
	GDM diagnosed	10.5%	44	183	15.9%	(11.5%, 22.0%)	1.73	(1.25, 2.39)	0.0009
	GDM not screened	33.4%	67	580	9.1%	(6.9%, 12.1%)	0.99	(0.75, 1.31)	0.96
Whether premature	No prematurity	95.2%	215	1,651	9.2%	(6.7%, 12.7%)	1.00		
	Prem: 32-35 weeks	3.1%	5	53	6.1%	(2.6%, 14.1%)	0.66	(0.28, 1.54)	0.34
	Prem: <32 weeks	1.8%	4	31	7.7%	(3.0%, 19.5%)	0.83	(0.33, 2.12)	0.70
Age of mother (years)	Mother <25	29.9%	67	490	9.2%	(6.7%, 12.7%)	1.00		
	Mother 25-35	54.5%	122	1,035	8.6%	(6.5%, 11.3%)	0.93	(0.71, 1.23)	0.62
	Mother 35+	15.6%	35	210	11.5%	(7.9%, 16.6%)	1.24	(0.86, 1.80)	0.25
SEIFA band [3]	High/middle SEIFA	58.0%	130	1,132	9.2%	(6.7%, 12.7%)	1.00		
	Low SEIFA	42.0%	94	603	11.4%	(9.0%, 14.6%)	1.24	(0.97, 1.59)	0.083
Smoking	Current non-smoker	63.4%	142	1,289	9.2%	(6.7%, 12.7%)	1.00		
	Current smoker	36.6%	82	446	11.1%	(9.9%, 12.3%)	1.20	(1.08, 1.34)	0.0008
Hypertension and/or Pre-eclampsia	No HT/P-Ecl	95.5%	214	1,618	9.2%	(6.7%, 12.7%)	1.00		
	HT/P-Ecl	4.5%	10	117	5.0%	(2.8%, 8.8%)	0.54	(0.31, 0.95)	0.033

Table S7: The effect of BMI status at conception on the rate of gestational weight gain above recommendations

Predictor	Predictor groupings	Group % BMI [4]	n	N	% in group with condition [5]	95%CI	IRR [5]	95%CI	P-value
				Outcome: Gestational weight gain above recommendations					
BMI (kg.m^2) at conception [1]	<18.5	3.7%	8	64	14.9%	(7.6%, 29.0%)	0.30	(0.15, 0.58)	0.0004
	18.5-25	40.6%	301	704	49.9%	(43.9%, 56.7%)	1.00		
	25-30	27.0%	231	469	57.3%	(50.6%, 64.8%)	1.15	(1.02, 1.30)	0.028
	30-35	14.1%	111	244	52.7%	(44.9%, 61.8%)	1.06	(0.90, 1.24)	0.50
	35+	14.6%	106	254	48.6%	(40.9%, 57.8%)	0.98	(0.82, 1.16)	0.78
DM screening status & result [2]	GDM excluded	56.0%	487	972	49.9%	(43.9%, 56.7%)	1.00		
	GDM diagnosed	10.5%	66	183	35.1%	(28.5%, 43.3%)	0.70	(0.57, 0.87)	0.0010
	GDM not screened	33.4%	204	580	36.7%	(32.3%, 41.6%)	0.73	(0.65, 0.83)	<0.0001
Whether premature	No prematurity	95.2%	733	1,651	49.9%	(43.9%, 56.7%)	1.00		
	Prem: 32-35 weeks	3.1%	22	53	47.9%	(34.8%, 65.9%)	0.96	(0.70, 1.32)	0.80
	Prem: <32 weeks	1.8%	2	31	8.9%	(2.3%, 34.4%)	0.18	(0.05, 0.69)	0.013
Age of mother (years)	Mother <25	29.5%	223	490	49.9%	(43.9%, 56.7%)	1.00		
	Mother 25-35	59.2%	448	1,035	46.7%	(41.5%, 52.5%)	0.94	(0.83, 1.05)	0.27
	Mother 35+	11.4%	86	210	45.9%	(38.0%, 55.4%)	0.92	(0.76, 1.11)	0.39
SEIFA band [3]	High/middle SEIFA	64.2%	486	1,132	49.9%	(43.9%, 56.7%)	1.00		
	Low SEIFA	35.8%	271	603	51.6%	(46.2%, 57.7%)	1.04	(0.93, 1.16)	0.54
Smoking	Current non-smoker	75.4%	571	1,289	49.9%	(43.9%, 56.7%)	1.00		
	Current smoker	24.6%	186	446	49.4%	(46.9%, 52.2%)	0.99	(0.94, 1.05)	0.75
Hypertension and/or Pre-eclampsia	No HT/P-Ecl	90.8%	687	1,618	49.9%	(43.9%, 56.7%)	1.00		
	HT/P-Ecl	9.2%	70	117	69.4%	(59.5%, 81.0%)	1.39	(1.19, 1.62)	<0.0001

Table S8: The effect of BMI status at conception and GWG at delivery on the rate of macrosomia

Predictor	Predictor groupings	Group % BMI [4]	n	N	% in group with condition [5]	95%CI	IRR [5]	95%CI	P-value
				Outcome: Macrosomia (birth weight >4000g)					
BMI (kg.m²) at conception [1]	<18.5	3.7%	0	64	0.0%	(0.0%, 0.0%)	0.00	(0.00, 0.00)	<0.0001
	18.5-25	40.6%	77	704	9.0%	(6.4%, 12.7%)	1.00		
	25-30	27.0%	89	469	14.3%	(10.8%, 18.9%)	1.59	(1.20, 2.10)	0.0011
	30-35	14.1%	37	244	12.0%	(8.3%, 17.3%)	1.33	(0.92, 1.92)	0.12
	35+	14.6%	47	254	16.0%	(11.4%, 22.5%)	1.77	(1.26, 2.49)	0.0010
Birth GWG versus recommendations [1]	Within	43.5%	82	754	9.0%	(6.4%, 12.7%)	1.00		
	Above	43.6%	149	757	14.9%	(11.7%, 19.1%)	1.66	(1.30, 2.12)	<0.0001
	Below	12.9%	19	224	6.4%	(4.0%, 10.2%)	0.71	(0.44, 1.13)	0.15
DM screening status & result [2]	GDM excluded	56.0%	147	972	9.0%	(6.4%, 12.7%)	1.00		
	GDM diagnosed	10.5%	34	183	11.2%	(8.0%, 15.8%)	1.25	(0.89, 1.75)	0.21
	GDM not screened	33.4%	69	580	8.1%	(6.3%, 10.5%)	0.90	(0.70, 1.17)	0.44
Whether premature	No prematurity	95.2%	250	1,651	9.0%	(6.4%, 12.7%)	1.00		
	Prem: 32-35 weeks	3.1%	0	53	0.0%	(0.0%, 0.0%)	0.00	(0.00, 0.00)	<0.0001
	Prem: <32 weeks	1.8%	0	31	0.0%	(0.0%, 0.0%)	0.00	(0.00, 0.00)	<0.0001
Age of mother (years)	Mother <25	28.2%	63	490	9.0%	(6.4%, 12.7%)	1.00		
	Mother 25-35	59.7%	158	1,035	9.5%	(7.3%, 12.5%)	1.06	(0.81, 1.38)	0.67
	Mother 35+	12.1%	29	210	8.8%	(5.9%, 13.1%)	0.98	(0.66, 1.46)	0.91
SEIFA band [3]	High/middle SEIFA	65.2%	159	1,132	9.0%	(6.4%, 12.7%)	1.00		
	Low SEIFA	34.8%	91	603	10.1%	(8.0%, 12.8%)	1.12	(0.88, 1.42)	0.36
Smoking	Current non-smoker	74.3%	208	1,289	9.0%	(6.4%, 12.7%)	1.00		
	Current smoker	25.7%	42	446	7.3%	(6.4%, 8.4%)	0.81	(0.71, 0.93)	0.0024
Hypertension and/or Pre-eclampsia	No HT/P-Ecl	93.3%	242	1,618	9.0%	(6.4%, 12.7%)	1.00		
	HT/P-Ecl	6.7%	8	117	3.2%	(1.7%, 6.2%)	0.36	(0.18, 0.69)	0.0023

Table S9: The effect of BMI status at conception and GWG at delivery on the rate of birth weight less than 2500g

Predictor	Predictor groupings	Group % BMI [4]	n	N	% in group with condition [5]	95%CI	IRR [5]	95%CI	P-value
		Outcome: Microsomia (birth weight <2500g)							
BMI (kg.m^2) at conception [1]	<18.5	3.6%	6	61	3.4%	(1.9%, 6.2%)	0.73	(0.41, 1.32)	0.30
	18.5-25	40.6%	49	691	4.7%	(2.9%, 7.7%)	1.00		
	25-30	27.1%	27	462	5.1%	(3.4%, 7.6%)	1.08	(0.73, 1.61)	0.71
	30-35	14.0%	13	239	5.7%	(3.2%, 10.0%)	1.21	(0.69, 2.11)	0.52
	35+	14.7%	12	251	4.0%	(2.2%, 7.2%)	0.85	(0.47, 1.53)	0.59
Birth GWG versus recommendations [1]	Within	42.8%	54	729	3.9%	(2.9%, 7.7%)	1.00		
	Above	44.3%	31	755	2.3%	(1.4%, 3.6%)	0.60	(0.41, 0.87)	0.0068
	Below	12.9%	22	220	5.9%	(3.4%, 9.6%)	1.54	(1.00, 2.36)	0.050
DM screening status & result [2]	GDM excluded	56.8%	58	968	4.7%	(2.9%, 7.7%)	1.00		
	GDM diagnosed	10.6%	10	181	3.5%	(1.9%, 6.3%)	0.73	(0.40, 1.34)	0.32
	GDM not screened	32.6%	39	555	5.3%	(3.7%, 7.5%)	1.13	(0.79, 1.60)	0.51
Whether premature	No prematurity	96.9%	65	1,651	4.7%	(2.9%, 7.7%)	1.00		
	Prem: 32-35 weeks	3.1%	42	53	85.6%	(61.5%, 100%)	18.17	(13.1, 25.3)	<0.0001
	(Prem: <32 weeks excluded in this model)		31	31	100%				
Age of mother (years)	Mother <25	28.2%	45	481	4.7%	(2.9%, 7.7%)	1.00		
	Mother 25-35	59.9%	52	1,021	3.6%	(2.5%, 5.3%)	0.77	(0.53, 1.12)	0.17
	Mother 35+	11.9%	10	202	3.1%	(1.8%, 5.5%)	0.67	(0.38, 1.18)	0.16
SEIFA band [3]	High/middle SEIFA	65.4%	61	1,115	4.7%	(2.9%, 7.7%)	1.00		
	Low SEIFA	34.6%	46	589	5.1%	(3.6%, 7.2%)	1.08	(0.76, 1.54)	0.66
Smoking	Current non-smoker	74.7%	60	1,273	4.7%	(2.9%, 7.7%)	1.00		
	Current smoker	25.3%	47	431	5.9%	(5.1%, 6.8%)	1.25	(1.09, 1.44)	0.0020
Hypertension and/or Pre-eclampsia	No HT/P-Ecl	93.2%	95	1,588	4.7%	(2.9%, 7.7%)	1.00		
	HT/P-Ecl	6.8%	12	116	8.1%	(4.7%, 14.0%)	1.73	(1.01, 2.97)	0.047

Tables S10 to S16 show the associations between maternal outcomes and predictors of those outcomes

Table S10: The effect of BMI status at conception and GWG at delivery on the rate of gestational diabetes mellitus in this pregnancy or prior diabetes mellitus

Predictor	Predictor groupings	Group % BMI [4]	n	N	% in group with condition [5]	95%CI	IRR [5]	95%CI	P-value
				Outcome: Gestational diabetes or prior diabetes mellitus					
BMI (kg.m^2) at conception [1]	<18.5	3.7%	1	64	1.4%	(0.2%, 10.4%)	0.33	(0.04, 2.46)	0.28
	18.5-25	40.6%	32	704	4.2%	(2.7%, 6.5%)	1.00		
	25-30	27.0%	47	469	9.5%	(6.2%, 14.6%)	2.25	(1.46, 3.47)	0.0002
	30-35	14.1%	31	244	11.4%	(7.1%, 18.2%)	2.70	(1.68, 4.33)	<0.0001
	35+	14.6%	72	254	23.3%	(15.6%, 34.8%)	5.53	(3.70, 8.25)	<0.0001
Birth GWG versus recommendations [1]	Within	43.5%	73	754	4.2%	(2.7%, 6.5%)	1.00		
	Above	43.6%	66	757	3.3%	(2.4%, 4.5%)	0.79	(0.58, 1.08)	0.14
	Below	12.9%	44	224	6.2%	(4.4%, 8.6%)	1.47	(1.05, 2.05)	0.024
Whether premature	No prematurity	95.2%	173	1,651	4.2%	(2.7%, 6.5%)	1.00		
	Prem: 32-35 weeks	3.1%	8	53	7.0%	(3.7%, 13.3%)	1.66	(0.87, 3.16)	0.13
	Prem: <32 weeks	1.8%	2	31	2.7%	(0.7%, 11.1%)	0.65	(0.16, 2.65)	0.55
Age of mother (years)	Mother <25	22.4%	41	490	4.2%	(2.7%, 6.5%)	1.00		
	Mother 25-35	55.2%	101	1,035	4.4%	(3.1%, 6.2%)	1.04	(0.74, 1.46)	0.81
	Mother 35+	22.4%	41	210	7.9%	(5.4%, 11.5%)	1.88	(1.29, 2.74)	0.0010
SEIFA band [3]	High/middle SEIFA	66.7%	122	1,132	4.2%	(2.7%, 6.5%)	1.00		
	Low SEIFA	33.3%	61	603	3.7%	(2.8%, 4.9%)	0.88	(0.66, 1.16)	0.36
Smoking	Current non-smoker	74.9%	137	1,289	4.2%	(2.7%, 6.5%)	1.00		
	Current smoker	25.1%	46	446	4.2%	(3.7%, 4.8%)	1.00	(0.88, 1.14)	0.96
Hypertension and/or Pre-eclampsia	No HT/P-Ecl	87.4%	160	1,618	4.2%	(2.7%, 6.5%)	1.00		
	HT/P-Ecl	12.6%	23	117	6.3%	(4.4%, 9.0%)	1.50	(1.05, 2.14)	0.025

Table S11: The effect of BMI status at conception and GWG at delivery on the rate of maternal hypertension and/or pre-eclampsia during pregnancy

Predictor	Predictor groupings	Group % BMI [4]	n	N	% in group with condition [5]	95%CI	IRR [5]	95%CI	P-value
Outcome: Hypertension of pregnancy or Pre-eclampsia									
BMI (kg.m^2) at conception [1]	<18.5	3.7%	2	64	4.1%	(0.9%, 18.3%)	0.99	(0.22, 4.42)	0.99
	18.5-25	40.6%	31	704	4.1%	(2.3%, 7.3%)	1.00		
	25-30	27.0%	30	469	5.2%	(3.1%, 8.8%)	1.26	(0.75, 2.12)	0.38
	30-35	14.1%	18	244	5.5%	(3.1%, 9.9%)	1.34	(0.75, 2.38)	0.32
	35+	14.6%	36	254	10.8%	(6.5%, 18.0%)	2.62	(1.58, 4.35)	0.0002
Birth GWG versus recommendations [1]	Within	31.6%	37	754	4.1%	(2.3%, 7.3%)	1.00		
	Above	59.8%	70	757	8.6%	(5.7%, 12.8%)	2.07	(1.39, 3.10)	0.0004
	Below	8.5%	10	224	2.9%	(1.4%, 5.7%)	0.69	(0.35, 1.37)	0.29
DM screening status & result [2]	GDM excluded	55.6%	65	972	4.1%	(2.3%, 7.3%)	1.00		
	GDM diagnosed	19.7%	23	183	6.1%	(3.8%, 9.9%)	1.49	(0.93, 2.38)	0.10
	GDM not screened	24.8%	29	580	3.5%	(2.2%, 5.4%)	0.84	(0.54, 1.31)	0.44
Whether premature	No prematurity	93.2%	109	1,651	4.1%	(2.3%, 7.3%)	1.00		
	Prem: 32-35 weeks	6.0%	7	53	12.0%	(5.6%, 26.1%)	2.91	(1.34, 6.31)	0.0067
	Prem: <32 weeks	0.9%	1	31	6.0%	(0.9%, 39.4%)	1.45	(0.22, 9.53)	0.70
Age of mother (years)	Mother <25	25.6%	30	490	4.1%	(2.3%, 7.3%)	1.00		
	Mother 25-35	63.2%	74	1,035	4.1%	(2.6%, 6.3%)	0.98	(0.63, 1.53)	0.94
	Mother 35+	11.1%	13	210	3.3%	(1.7%, 6.5%)	0.81	(0.41, 1.58)	0.53
SEIFA band [3]	High/middle SEIFA	59.8%	70	1,132	4.1%	(2.3%, 7.3%)	1.00		
	Low SEIFA	40.2%	47	603	4.8%	(3.3%, 7.0%)	1.17	(0.80, 1.70)	0.42
Smoking	Current non-smoker	82.9%	97	1,289	4.1%	(2.3%, 7.3%)	1.00		
	Current smoker	17.1%	20	446	3.4%	(2.7%, 4.2%)	0.82	(0.66, 1.02)	0.071

Table S12: The effect of BMI status at conception and GWG at delivery on the rate of maternal mental health disorders during pregnancy and delivery period

Predictor	Predictor groupings	Group % BMI [4]	n	N	% in group with condition [5]	95%CI	IRR [5]	95%CI	P-value
		Outcome: Anxiety, depression, postnatal depression; during pregnancy, post-delivery, or pre-existing							
BMI (kg.m^2) at conception [1]	<18.5	3.7%	31	64	39.4%	(30.6%, 50.7%)	1.32	(1.02, 1.70)	0.033
	18.5-25	40.6%	227	704	29.9%	(25.3%, 35.3%)	1.00		
	25-30	27.0%	162	469	32.9%	(28.0%, 38.6%)	1.10	(0.94, 1.29)	0.24
	30-35	14.1%	107	244	41.5%	(34.8%, 49.6%)	1.39	(1.17, 1.66)	0.0003
	35+	14.6%	122	254	45.2%	(38.1%, 53.6%)	1.51	(1.27, 1.79)	<0.0001
Birth GWG versus recommendations [1]	Within	43.5%	268	754	29.9%	(25.3%, 35.3%)	1.00		
	Above	43.6%	278	757	31.2%	(27.3%, 35.7%)	1.05	(0.92, 1.19)	0.51
	Below	12.9%	103	224	33.8%	(28.5%, 40.1%)	1.13	(0.96, 1.34)	0.15
DM screening status & result [2]	GDM excluded	56.0%	346	972	29.9%	(25.3%, 35.3%)	1.00		
	GDM diagnosed	10.5%	72	183	30.7%	(25.0%, 37.6%)	1.03	(0.84, 1.26)	0.80
	GDM not screened	33.4%	231	580	33.2%	(29.1%, 37.8%)	1.11	(0.98, 1.26)	0.11
Whether premature	No prematurity	95.2%	604	1,651	29.9%	(25.3%, 35.3%)	1.00		
	Prem: 32-35 weeks	3.1%	27	53	39.7%	(29.7%, 52.9%)	1.33	(0.99, 1.77)	0.054
	Prem: <32 weeks	1.8%	18	31	41.2%	(29.6%, 57.3%)	1.38	(0.99, 1.92)	0.056
Age of mother (years)	Mother <25	33.9%	220	490	29.9%	(25.3%, 35.3%)	1.00		
	Mother 25-35	57.0%	370	1,035	26.1%	(23.0%, 29.7%)	0.87	(0.77, 0.99)	0.038
	Mother 35+	9.1%	59	210	20.1%	(15.9%, 25.4%)	0.67	(0.53, 0.85)	0.0009
SEIFA band [3]	High/middle SEIFA	57.6%	374	1,132	29.9%	(25.3%, 35.3%)	1.00		
	Low SEIFA	42.4%	275	603	36.2%	(32.1%, 40.9%)	1.21	(1.07, 1.37)	0.0020
Smoking	Current non-smoker	62.6%	406	1,289	29.9%	(25.3%, 35.3%)	1.00		
	Current smoker	37.4%	243	446	36.2%	(34.4%, 38.2%)	1.21	(1.15, 1.28)	<0.0001
Hypertension and/or Pre-eclampsia	No HT/P-Ecl	94.3%	612	1,618	29.9%	(25.3%, 35.3%)	1.00		
	HT/P-Ecl	5.7%	37	117	24.4%	(18.6%, 31.9%)	0.82	(0.62, 1.07)	0.14

Table S13: The effect of BMI status at conception and GWG at delivery on the rate of delivery by Caesarean section

Predictor	Predictor groupings	Group % BMI [4]	n	N	% in group with condition [5]	95%CI	IRR [5]	95%CI	P-value
		Outcome: Caesarean section delivery							
BMI (kg.m^2) at conception [1]	<18.5	3.7%	13	64	16.9%	(10.2%, 27.9%)	0.64	(0.39, 1.05)	0.080
	18.5-25	40.6%	243	704	26.5%	(21.9%, 31.9%)	1.00		
	25-30	27.0%	175	469	27.7%	(23.7%, 32.3%)	1.05	(0.90, 1.22)	0.57
	30-35	14.1%	88	244	26.9%	(22.2%, 32.7%)	1.02	(0.84, 1.23)	0.86
	35+	14.6%	121	254	33.4%	(28.3%, 39.5%)	1.26	(1.07, 1.49)	0.0065
Birth GWG versus recommendations [1]	Within	43.5%	252	754	26.5%	(21.9%, 31.9%)	1.00		
	Above	43.6%	313	757	32.8%	(28.7%, 37.5%)	1.24	(1.08, 1.42)	0.0017
	Below	12.9%	75	224	25.9%	(21.0%, 31.9%)	0.98	(0.79, 1.21)	0.84
DM screening status & result [2]	GDM excluded	56.0%	346	972	26.5%	(21.9%, 31.9%)	1.00		
	GDM diagnosed	10.5%	93	183	34.8%	(29.3%, 41.3%)	1.32	(1.11, 1.56)	0.0018
	GDM not screened	33.4%	201	580	26.5%	(23.0%, 30.5%)	1.00	(0.87, 1.15)	0.97
Whether birth premature	No prematurity	95.2%	591	1,651	26.5%	(21.9%, 31.9%)	1.00		
	Prem: 32-35 weeks	3.1%	33	53	47.0%	(38.0%, 58.3%)	1.78	(1.43, 2.20)	<0.0001
	Prem: <32 weeks	1.8%	16	31	45.2%	(31.7%, 64.4%)	1.71	(1.20, 2.43)	0.0031
Age of mother (years)	Mother <25	24.4%	156	490	26.5%	(21.9%, 31.9%)	1.00		
	Mother 25-35	61.1%	391	1,035	30.1%	(25.9%, 35.0%)	1.14	(0.98, 1.32)	0.092
	Mother 35+	14.5%	93	210	33.9%	(27.7%, 41.4%)	1.28	(1.05, 1.57)	0.016
SEIFA band [3]	High/middle SEIFA	65.8%	421	1,132	26.5%	(21.9%, 31.9%)	1.00		
	Low SEIFA	34.2%	219	603	26.7%	(23.4%, 30.4%)	1.01	(0.89, 1.15)	0.90
Smoking	Current non-smoker	78.1%	500	1,289	26.5%	(21.9%, 31.9%)	1.00		
	Current smoker	21.9%	140	446	24.3%	(22.7%, 26.0%)	0.92	(0.86, 0.98)	0.011
Hypertension and/or Pre-eclampsia	No HT/P-Ecl	91.3%	584	1,618	26.5%	(21.9%, 31.9%)	1.00		
	HT/P-Ecl	8.8%	56	117	30.1%	(24.7%, 36.6%)	1.14	(0.93, 1.38)	0.20

Table S14: The effect of BMI status at conception and GWG at delivery on the rate of instrumental delivery

Predictor	Predictor groupings	Group % BMI [4]	n	N	% in group with condition [5]	95%CI	IRR [5]	95%CI	P-value
			Outcome: Instrumental delivery						
BMI (kg.m^2) at conception [1]	<18.5	3.7%	5	64	9.2%	(3.8%, 22.4%)	1.05	(0.43, 2.57)	0.91
	18.5-25	40.6%	62	704	8.7%	(5.4%, 14.1%)	1.00		
	25-30	27.0%	39	469	7.9%	(5.4%, 11.5%)	0.90	(0.62, 1.32)	0.59
	30-35	14.1%	16	244	6.4%	(3.8%, 10.7%)	0.73	(0.44, 1.22)	0.23
	35+	14.6%	10	254	4.1%	(2.1%, 8.0%)	0.47	(0.24, 0.91)	0.025
Birth GWG versus recommendations [1]	Within	43.5%	43	754	8.7%	(5.4%, 14.1%)	1.00		
	Above	43.6%	75	757	14.6%	(10.0%, 21.5%)	1.68	(1.14, 2.46)	0.0083
	Below	12.9%	14	224	11.9%	(6.5%, 21.9%)	1.37	(0.75, 2.51)	0.31
DM screening status & result [2]	GDM excluded	56.0%	86	972	8.7%	(5.4%, 14.1%)	1.00		
	GDM diagnosed	10.5%	7	183	5.1%	(2.3%, 11.1%)	0.58	(0.27, 1.27)	0.17
	GDM not screened	33.4%	39	580	7.4%	(5.1%, 10.7%)	0.85	(0.59, 1.22)	0.38
Whether premature	No prematurity	95.2%	129	1,651	8.7%	(5.4%, 14.1%)	1.00		
	Prem: 32-35 weeks	3.1%	2	53	4.5%	(1.2%, 17.0%)	0.52	(0.14, 1.94)	0.33
	Prem: <32 weeks	1.8%	1	31	6.4%	(0.9%, 45.3%)	0.73	(0.10, 5.18)	0.75
Age of mother (years)	Mother <25	34.1%	45	490	8.7%	(5.4%, 14.1%)	1.00		
	Mother 25-35	59.1%	78	1,035	6.6%	(4.7%, 9.3%)	0.75	(0.53, 1.07)	0.11
	Mother 35+	6.8%	9	210	4.2%	(2.1%, 8.5%)	0.48	(0.24, 0.97)	0.040
SEIFA band [3]	High/middle SEIFA	67.4%	89	1,132	8.7%	(5.4%, 14.1%)	1.00		
	Low SEIFA	32.6%	43	603	8.6%	(6.0%, 12.3%)	0.98	(0.68, 1.41)	0.93
Smoking	Current non-smoker	87.1%	115	1,289	8.7%	(5.4%, 14.1%)	1.00		
	Current smoker	12.9%	17	446	6.0%	(4.8%, 7.5%)	0.69	(0.55, 0.85)	0.0007
Hypertension and/or Pre-eclampsia	No HT/P-Ecl	90.9%	120	1,618	8.7%	(5.4%, 14.1%)	1.00		
	HT/P-Ecl	9.1%	12	117	11.9%	(6.7%, 21.1%)	1.37	(0.77, 2.42)	0.28

Table S15: The effect of BMI status at conception and GWG at delivery on the rate of Moderate-to-severe genital trauma during delivery

Predictor	Predictor groupings	Group % BMI [4]	n	N	% in group with condition [5]	95%CI	IRR [5]	95%CI	P-value
Outcome: Moderate-to-severe genital trauma during delivery									
BMI (kg.m^2) at conception [1]	<18.5	3.7%	23	64	49.4%	(35.3%, 69.2%)	1.07	(0.77, 1.50)	0.69
	18.5-25	40.6%	247	704	46.1%	(38.9%, 54.7%)	1.00		
	25-30	27.0%	149	469	41.1%	(34.9%, 48.4%)	0.89	(0.76, 1.05)	0.17
	30-35	14.1%	80	244	43.3%	(35.2%, 53.2%)	0.94	(0.76, 1.15)	0.55
	35+	14.6%	66	254	36.4%	(28.7%, 46.1%)	0.79	(0.62, 1.00)	0.050
Birth GWG versus recommendations [1]	Within	43.5%	236	754	46.1%	(38.9%, 54.7%)	1.00		
	Above	43.6%	266	757	49.3%	(42.6%, 56.9%)	1.07	(0.92, 1.23)	0.37
	Below	12.9%	63	224	44.0%	(34.8%, 55.5%)	0.95	(0.75, 1.20)	0.69
DM screening status & result [2]	GDM excluded	56.0%	357	972	46.1%	(38.9%, 54.7%)	1.00		
	GDM diagnosed	10.5%	46	183	36.5%	(27.8%, 47.7%)	0.79	(0.60, 1.03)	0.087
	GDM not screened	33.4%	162	580	37.0%	(31.8%, 43.1%)	0.80	(0.69, 0.94)	0.0049
Whether premature	No prematurity	95.2%	556	1,651	46.1%	(38.9%, 54.7%)	1.00		
	Prem: 32-35 weeks	3.1%	9	53	24.1%	(13.3%, 43.4%)	0.52	(0.29, 0.94)	0.031
	Prem: <32 weeks	1.8%	0	31	0.0%	(0.0%, 0.0%)	0.00	(0.00, 0.00)	<0.0001
Age of mother (years)	Mother <25	32.0%	181	490	46.1%	(38.9%, 54.7%)	1.00		
	Mother 25-35	60.0%	339	1,035	38.2%	(33.1%, 44.1%)	0.83	(0.72, 0.96)	0.010
	Mother 35+	8.0%	45	210	26.8%	(20.3%, 35.4%)	0.58	(0.44, 0.77)	0.0001
SEIFA band [3]	High/middle SEIFA	67.4%	381	1,132	46.1%	(38.9%, 54.7%)	1.00		
	Low SEIFA	32.6%	184	603	43.6%	(37.7%, 50.4%)	0.94	(0.82, 1.09)	0.44
Smoking	Current non-smoker	81.2%	459	1,289	46.1%	(38.9%, 54.7%)	1.00		
	Current smoker	18.8%	106	446	39.0%	(36.1%, 42.1%)	0.84	(0.78, 0.91)	<0.0001
Hypertension and/or Pre-eclampsia	No HT/P-Ecl	93.6%	529	1,618	46.1%	(38.9%, 54.7%)	1.00		
	HT/P-Ecl	6.4%	36	117	43.2%	(32.8%, 56.9%)	0.94	(0.71, 1.23)	0.64

Table S16: The effect of BMI status at conception and GWG at delivery on the rate of post-partum haemorrhage

Predictor	Predictor groupings	Group % BMI [4]	n	N	% in group with condition [5]	95%CI	IRR [5]	95%CI	P-value
		Outcome: Post-partum haemorrhage							
BMI (kg.m^2) at conception [1]	<18.5	3.7%	12	64	14.4%	(8.4%, 24.5%)	1.07	(0.63, 1.83)	0.79
	18.5-25	40.6%	138	704	13.4%	(10.1%, 17.7%)	1.00		
	25-30	27.0%	95	469	13.3%	(10.5%, 16.9%)	1.00	(0.79, 1.26)	0.97
	30-35	14.1%	67	244	18.2%	(14.1%, 23.4%)	1.36	(1.05, 1.74)	0.019
	35+	14.6%	60	254	15.0%	(11.4%, 19.8%)	1.12	(0.85, 1.48)	0.42
Birth GWG versus recommendations [1]	Within	43.5%	137	754	13.4%	(10.1%, 17.7%)	1.00		
	Above	43.6%	187	757	18.3%	(14.9%, 22.4%)	1.36	(1.12, 1.67)	0.0025
	Below	12.9%	48	224	15.2%	(11.3%, 20.4%)	1.13	(0.84, 1.52)	0.42
DM screening status & result [2]	GDM excluded	56.0%	216	972	13.4%	(10.1%, 17.7%)	1.00		
	GDM diagnosed	10.5%	47	183	14.6%	(11.0%, 19.4%)	1.09	(0.82, 1.45)	0.54
	GDM not screened	33.4%	109	580	11.8%	(9.5%, 14.5%)	0.88	(0.71, 1.08)	0.22
Whether premature	No prematurity	95.2%	350	1,651	13.4%	(10.1%, 17.7%)	1.00		
	Prem: 32-35 weeks	3.1%	14	53	17.8%	(11.3%, 28.0%)	1.33	(0.84, 2.09)	0.23
	Prem: <32 weeks	1.8%	8	31	19.1%	(10.1%, 36.1%)	1.42	(0.75, 2.70)	0.28
Age of mother (years)	Mother <25	21.8%	81	490	13.4%	(10.1%, 17.7%)	1.00		
	Mother 25-35	61.8%	230	1,035	18.0%	(14.3%, 22.7%)	1.35	(1.07, 1.70)	0.012
	Mother 35+	16.4%	61	210	23.0%	(17.1%, 31.1%)	1.72	(1.27, 2.32)	0.0004
SEIFA band [3]	High/middle SEIFA	65.6%	244	1,132	13.4%	(10.1%, 17.7%)	1.00		
	Low SEIFA	34.4%	128	603	13.6%	(11.2%, 16.5%)	1.02	(0.84, 1.23)	0.87
Smoking	Current non-smoker	76.1%	283	1,289	13.4%	(10.1%, 17.7%)	1.00		
	Current smoker	23.9%	89	446	13.1%	(11.9%, 14.3%)	0.97	(0.89, 1.07)	0.59
Hypertension and/or Pre-eclampsia	No HT/P-Ecl	93.3%	347	1,618	13.4%	(10.1%, 17.7%)	1.00		
	HT/P-Ecl	6.7%	25	117	12.0%	(8.3%, 17.2%)	0.89	(0.62, 1.28)	0.54

Tables S17 to S18 show the associations between outcomes for the babies and predictors of those outcomes

Table S17: The effect of BMI status at conception and GWG at delivery on the rate of stillbirth

Predictor	Predictor groupings	Group % BMI [4]	n	N	% in group with condition [5]	95%CI	IRR [5]	95%CI	P-value
		Outcome: Stillbirth							
BMI (kg.m²) at conception [1]	<18.5	3.7%	1	64	0.10%	(0.02%, 0.45%)	0.94	(0.21, 4.19)	0.93
	18.5-25	40.6%	6	704	0.11%	(0.01%, 0.85%)	1.00		
	25-30	27.0%	2	469	0.14%	(0.03%, 0.66%)	1.28	(0.26, 6.19)	0.76
	30-35	14.1%	3	244	0.14%	(0.05%, 0.42%)	1.33	(0.45, 3.92)	0.61
	35+	14.6%	1	254	0.07%	(0.02%, 0.31%)	0.68	(0.16, 2.92)	0.61
Birth GWG versus recommendations [1]	Within	43.5%	12	754	0.11%	(0.01%, 0.85%)	1.00		
	Above	43.6%	1	757	0.03%	(0.01%, 0.18%)	0.32	(0.06, 1.68)	0.18
	Below	12.9%	0	224	0.00%	(0.00%, 0.00%)	0.00	(0.00, 0.00)	<0.0001
DM screening status & result [2]	GDM excluded	56.0%	2	972	0.11%	(0.01%, 0.85%)	1.00		
	GDM diagnosed	10.5%	0	183	0.05%	(0.02%, 0.13%)	0.46	(0.18, 1.17)	0.10
	GDM not screened	33.4%	11	580	0.00%	(0.00%, 0.00%)	0.00	(0.00, 0.00)	<0.0001
Whether premature	No prematurity	95.2%	2	1,651	0.11%	(0.01%, 0.85%)	1.00		
	Prem: 32-35 weeks	3.1%	0	53	0.00%	(0.00%, 0.00%)	0.00	(0.00, 0.00)	<0.0001
	Prem: <32 weeks	1.8%	11	31	24.8%	(4.6%, 100.0%)	230.6	(42.7, 1245)	<0.0001
Age of mother (years)	Mother <25	28.2%	3	490	0.11%	(0.01%, 0.85%)	1.00		
	Mother 25-35	59.7%	7	1,035	0.16%	(0.05%, 0.50%)	1.51	(0.49, 4.64)	0.47
	Mother 35+	12.1%	3	210	0.20%	(0.05%, 0.83%)	1.85	(0.44, 7.76)	0.40
SEIFA band [3]	High/middle SEIFA	65.2%	7	1,132	0.11%	(0.01%, 0.85%)	1.00		
	Low SEIFA	34.8%	6	603	0.18%	(0.07%, 0.48%)	1.65	(0.61, 4.44)	0.33
Smoking	Current non-smoker	74.3%	8	1,289	0.11%	(0.01%, 0.85%)	1.00		
	Current smoker	25.7%	5	446	0.11%	(0.08%, 0.15%)	1.00	(0.71, 1.40)	0.999
Hypertension and/or Pre-eclampsia	No HT/P-Ecl	93.3%	13	1,618	0.11%	(0.01%, 0.85%)	1.00		
	HT/P-Ecl	6.7%	0	117	0.00%	(0.00%, 0.00%)	0.00	(0.00, 0.00)	<0.0001

Table S18: The effect of BMI status at conception and GWG at delivery on the rate of prematurity before 32 weeks

Predictor	Predictor groupings	Group % BMI [4]	n	N	% in group with condition [5]	95%CI	IRR [5]	95%CI	P-value
				Outcome: Prematurity before 32 weeks					
BMI (kg.m^2) at conception [1]	<18.5	3.7%	3	64	4.61%	(1.25%, 16.9%)	2.01	(0.54, 7.38)	0.30
	18.5-25	40.6%	13	704	2.30%	(0.80%, 6.62%)	1.00		
	25-30	27.0%	7	469	2.14%	(0.83%, 5.49%)	0.93	(0.36, 2.39)	0.88
	30-35	14.1%	5	244	2.97%	(1.05%, 8.38%)	1.29	(0.46, 3.65)	0.63
	35+	14.6%	3	254	1.45%	(0.39%, 5.45%)	0.63	(0.17, 2.37)	0.50
Birth GWG versus recommendations [1]	Within	43.5%	25	754	2.30%	(0.80%, 6.62%)	1.00		
	Above	43.6%	2	757	0.19%	(0.04%, 0.79%)	0.08	(0.02, 0.34)	0.0006
	Below	12.9%	4	224	0.96%	(0.33%, 2.77%)	0.42	(0.15, 1.21)	0.11
Age of mother (years)	Mother <25	28.2%	9	490	2.30%	(0.80%, 6.62%)	1.00		
	Mother 25-35	59.7%	14	1,035	2.06%	(0.87%, 4.85%)	0.90	(0.38, 2.11)	0.80
	Mother 35+	12.1%	8	210	5.89%	(2.11%, 16.5%)	2.56	(0.92, 7.17)	0.073
SEIFA band [3]	High/middle SEIFA	65.2%	17	1,132	2.30%	(0.80%, 6.62%)	1.00		
	Low SEIFA	34.8%	14	603	3.38%	(1.70%, 6.69%)	1.47	(0.74, 2.91)	0.27
Smoking	Current non-smoker	74.3%	16	1,289	2.30%	(0.80%, 6.62%)	1.00		
	Current smoker	25.7%	15	446	3.50%	(2.60%, 4.73%)	1.52	(1.13, 2.06)	0.0057
Hypertension and/or Pre-eclampsia	No HT/P-Ecl	93.3%	30	1,618	2.30%	(0.80%, 6.62%)	1.00		
	HT/P-Ecl	6.7%	1	117	1.51%	(0.22%, 10.6%)	0.66	(0.09, 4.60)	0.67

There is extreme confounding between prematurity and lack of GDM screening: early prematurity occurs before GDM screening is completed.

Table S19: The effect of BMI status at conception and GWG at delivery on the rate of immediate admission to the Special Care Nursery

Predictor	Predictor groupings	Group % BMI [4]	n	N	% in group with condition [5]	95%CI	IRR [5]	95%CI	P-value
Outcome: Immediate admission to the Special Care Nursery									
BMI (kg.m^2) at conception [1]	<18.5	3.7%	11	64	13.5%	(7.8%, 23.2%)	1.00	(0.58, 1.73)	0.99
	18.5-25	40.6%	104	704	13.4%	(9.9%, 18.1%)	1.00		
	25-30	27.0%	77	469	15.1%	(11.6%, 19.7%)	1.13	(0.87, 1.47)	0.36
	30-35	14.1%	30	244	11.3%	(7.8%, 16.4%)	0.84	(0.58, 1.23)	0.37
	35+	14.6%	43	254	13.0%	(9.3%, 18.2%)	0.97	(0.69, 1.36)	0.87
Birth GWG versus recommendations [1]	Within	43.5%	123	754	13.4%	(9.9%, 18.1%)	1.00		
	Above	43.6%	102	757	11.3%	(8.9%, 14.4%)	0.85	(0.66, 1.08)	0.17
	Below	12.9%	40	224	14.4%	(10.3%, 20.0%)	1.07	(0.77, 1.49)	0.68
DM screening status & result [2]	GDM excluded	56.0%	132	972	13.4%	(9.9%, 18.1%)	1.00		
	GDM diagnosed	10.5%	49	183	23.5%	(17.4%, 31.9%)	1.76	(1.30, 2.38)	0.0003
	GDM not screened	33.4%	84	580	13.3%	(10.4%, 17.1%)	0.99	(0.77, 1.27)	0.95
Whether birth premature	No prematurity	95.2%	220	1,651	13.4%	(9.9%, 18.1%)	1.00		
	Prem: 32-35 weeks	3.1%	35	53	58.9%	(45.6%, 76.0%)	4.39	(3.41, 5.67)	<0.0001
	Prem: <32 weeks	1.8%	10	31	29.9%	(17.0%, 52.5%)	2.23	(1.27, 3.92)	0.0053
Age of mother (years)	Mother <25	28.2%	85	490	13.4%	(9.9%, 18.1%)	1.00		
	Mother 25-35	59.7%	140	1,035	11.3%	(8.9%, 14.5%)	0.85	(0.66, 1.08)	0.18
	Mother 35+	12.1%	40	210	14.0%	(10.0%, 19.5%)	1.04	(0.74, 1.46)	0.81
SEIFA band [3]	High/middle SEIFA	65.2%	165	1,132	13.4%	(9.9%, 18.1%)	1.00		
	Low SEIFA	34.8%	100	603	14.5%	(11.5%, 18.2%)	1.08	(0.86, 1.36)	0.51
Smoking	Current non-smoker	74.3%	180	1,289	13.4%	(9.9%, 18.1%)	1.00		
	Current smoker	25.7%	85	446	14.5%	(13.1%, 16.0%)	1.08	(0.98, 1.19)	0.12
Hypertension and/or Pre-eclampsia	No HT/P-Ecl	93.3%	237	1,618	13.4%	(9.9%, 18.1%)	1.00		
	HT/P-Ecl	6.7%	28	117	19.6%	(13.9%, 27.7%)	1.46	(1.04, 2.06)	0.029

Table S20: The effect of BMI status at conception and GWG at delivery on the rate of immediate admission to the Neonatal Intensive Care Unit

Predictor	Predictor groupings	Group % BMI [4]	n	N	% in group with condition [5]	95%CI	IRR [5]	95%CI	P-value
Outcome: Immediate admission to the Neonatal Intensive Care Unit									
BMI (kg.m^2) at conception [1]	<18.5	3.7%	4	64	0.6%	(0.3%, 1.4%)	0.92	(0.41, 2.03)	0.83
	18.5-25	40.6%	24	704	0.7%	(0.3%, 1.7%)	1.00		
	25-30	27.0%	10	469	0.5%	(0.3%, 1.0%)	0.75	(0.40, 1.40)	0.36
	30-35	14.1%	7	244	0.8%	(0.3%, 1.9%)	1.10	(0.46, 2.64)	0.84
	35+	14.6%	12	254	1.1%	(0.7%, 1.8%)	1.54	(0.92, 2.58)	0.097
Birth GWG versus recommendations [1]	Within	43.5%	27	754	0.7%	(0.3%, 1.7%)	1.00		
	Above	43.6%	19	757	0.8%	(0.5%, 1.4%)	1.18	(0.70, 1.99)	0.53
	Below	12.9%	11	224	1.5%	(0.9%, 2.4%)	2.05	(1.24, 3.41)	0.0053
DM screening status & result [2]	GDM excluded	56.0%	23	972	0.7%	(0.3%, 1.7%)	1.00		
	GDM diagnosed	10.5%	8	183	1.0%	(0.5%, 2.0%)	1.42	(0.72, 2.82)	0.31
	GDM not screened	33.4%	26	580	0.9%	(0.5%, 1.6%)	1.30	(0.75, 2.25)	0.34
Whether premature	No prematurity	95.2%	18	1,651	0.7%	(0.3%, 1.7%)	1.00		
	Prem: 32-35 weeks	3.1%	25	53	32.5%	(18.7%, 56.6%)	45.96	(26.4, 79.9)	<0.0001
	Prem: <32 weeks	1.8%	14	31	29.1%	(14.1%, 60.0%)	41.08	(19.9, 84.7)	<0.0001
Age of mother (years)	Mother <25	28.2%	17	490	0.7%	(0.3%, 1.7%)	1.00		
	Mother 25-35	59.7%	30	1,035	0.7%	(0.4%, 1.3%)	1.04	(0.61, 1.77)	0.90
	Mother 35+	12.1%	10	210	0.9%	(0.4%, 1.9%)	1.26	(0.60, 2.63)	0.54
SEIFA band [3]	High/middle SEIFA	65.2%	34	1,132	0.7%	(0.3%, 1.7%)	1.00		
	Low SEIFA	34.8%	23	603	0.8%	(0.5%, 1.2%)	1.09	(0.69, 1.72)	0.73
Smoking	Current non-smoker	74.3%	37	1,289	0.7%	(0.3%, 1.7%)	1.00		
	Current smoker	25.7%	20	446	0.7%	(0.5%, 0.9%)	0.96	(0.77, 1.22)	0.76
Hypertension and/or Pre-eclampsia	No HT/P-Ecl	93.3%	53	1,618	0.7%	(0.3%, 1.7%)	1.00		
	HT/P-Ecl	6.7%	4	117	0.5%	(0.2%, 1.3%)	0.77	(0.33, 1.81)	0.55

Table S21: The effect of BMI status at conception and GWG at delivery on the rate of neonatal hypoglycaemia

Predictor	Predictor groupings	Group % BMI [4]	n	N	% in group with condition [5]	95%CI	IRR [5]	95%CI	P-value
		Outcome: Neonatal hypoglycaemia							
BMI (kg.m^2) at conception [1]	<18.5	3.7%	0	64	0.00%	(0.00%, 0.00%)	0.00	(0.00, 0.00)	<0.0001
	18.5-25	40.6%	19	704	1.42%	(0.73%, 2.78%)	1.00		
	25-30	27.0%	23	469	2.29%	(1.27%, 4.14%)	1.61	(0.89, 2.91)	0.12
	30-35	14.1%	14	244	2.38%	(1.15%, 4.90%)	1.67	(0.81, 3.44)	0.17
	35+	14.6%	19	254	2.14%	(1.14%, 4.02%)	1.50	(0.80, 2.82)	0.21
Birth GWG versus recommendations [1]	Within	43.5%	31	754	1.42%	(0.73%, 2.78%)	1.00		
	Above	43.6%	28	757	1.34%	(0.80%, 2.26%)	0.94	(0.56, 1.59)	0.82
	Below	12.9%	16	224	2.16%	(1.17%, 3.99%)	1.52	(0.82, 2.80)	0.19
DM screening status & result [2]	GDM excluded	56.0%	23	972	1.42%	(0.73%, 2.78%)	1.00		
	GDM diagnosed	10.5%	31	183	8.44%	(4.83%, 14.74%)	5.93	(3.39, 10.35)	<0.0001
	GDM not screened	33.4%	21	580	2.19%	(1.20%, 4.00%)	1.54	(0.84, 2.81)	0.16
Whether premature	No prematurity	95.2%	65	1,651	1.42%	(0.73%, 2.78%)	1.00		
	Prem: 32-35 weeks	3.1%	8	53	5.38%	(2.69%, 10.78%)	3.78	(1.89, 7.57)	0.0002
	Prem: <32 weeks	1.8%	2	31	2.73%	(0.65%, 11.36%)	1.91	(0.46, 7.98)	0.37
Age of mother (years)	Mother <25	28.2%	19	490	1.42%	(0.73%, 2.78%)	1.00		
	Mother 25-35	59.7%	48	1,035	1.67%	(1.01%, 2.77%)	1.17	(0.71, 1.94)	0.53
	Mother 35+	12.1%	8	210	0.92%	(0.39%, 2.19%)	0.65	(0.27, 1.54)	0.33
SEIFA band [3]	High/middle SEIFA	65.2%	46	1,132	1.42%	(0.73%, 2.78%)	1.00		
	Low SEIFA	34.8%	29	603	1.64%	(1.03%, 2.63%)	1.16	(0.72, 1.85)	0.55
Smoking	Current non-smoker	74.3%	53	1,289	1.42%	(0.73%, 2.78%)	1.00		
	Current smoker	25.7%	22	446	1.49%	(1.20%, 1.84%)	1.04	(0.84, 1.29)	0.70
Hypertension and/or Pre-eclampsia	No HT/P-Ecl	93.3%	66	1,618	1.42%	(0.73%, 2.78%)	1.00		
	HT/P-Ecl	6.7%	9	117	2.00%	(1.01%, 3.95%)	1.41	(0.71, 2.77)	0.33

Table S22: The effect of BMI status at conception and GWG at delivery on the rate of neonatal respiratory distress

Predictor	Predictor groupings	Group % BMI [4]	n	N	% in group with condition [5]	95%CI	IRR [5]	95%CI	P-value
				Outcome: Neonatal respiratory distress					
BMI ($kg.m^2$) at conception [1]	<18.5	3.7%	4	64	3.5%	(1.5%, 8.5%)	0.87	(0.36, 2.10)	0.76
	18.5-25	40.6%	36	704	4.1%	(2.5%, 6.7%)	1.00		
	25-30	27.0%	28	469	5.2%	(3.3%, 8.3%)	1.29	(0.81, 2.05)	0.29
	30-35	14.1%	12	244	4.2%	(2.2%, 7.8%)	1.03	(0.55, 1.93)	0.92
	35+	14.6%	16	254	4.8%	(2.7%, 8.5%)	1.19	(0.67, 2.11)	0.56
Birth GWG versus recommendations [1]	Within	43.5%	39	754	4.1%	(2.5%, 6.7%)	1.00		
	Above	43.6%	36	757	4.4%	(2.8%, 6.9%)	1.08	(0.69, 1.70)	0.74
	Below	12.9%	21	224	8.6%	(5.3%, 13.9%)	2.12	(1.30, 3.44)	0.0024
DM screening status & result [2]	GDM excluded	56.0%	53	972	4.1%	(2.5%, 6.7%)	1.00		
	GDM diagnosed	10.5%	13	183	4.2%	(2.3%, 7.6%)	1.04	(0.58, 1.87)	0.90
	GDM not screened	33.4%	30	580	3.0%	(1.9%, 4.6%)	0.74	(0.48, 1.15)	0.18
Whether premature	No prematurity	95.2%	68	1,651	4.1%	(2.5%, 6.7%)	1.00		
	Prem: 32-35 weeks	3.1%	17	53	33.4%	(21.2%, 52.7%)	8.25	(5.23, 13.0)	<0.0001
	Prem: <32 weeks	1.8%	11	31	42.7%	(23.4%, 78.0%)	10.54	(5.77, 19.3)	<0.0001
Age of mother (years)	Mother <25	28.2%	29	490	4.1%	(2.5%, 6.7%)	1.00		
	Mother 25-35	59.7%	54	1,035	3.9%	(2.6%, 5.9%)	0.97	(0.65, 1.46)	0.90
	Mother 35+	12.1%	13	210	3.6%	(1.9%, 6.9%)	0.89	(0.47, 1.70)	0.73
SEIFA band [3]	High/middle SEIFA	65.2%	68	1,132	4.1%	(2.5%, 6.7%)	1.00		
	Low SEIFA	34.8%	28	603	2.6%	(1.7%, 4.0%)	0.64	(0.42, 0.98)	0.039
Smoking	Current non-smoker	74.3%	63	1,289	4.1%	(2.5%, 6.7%)	1.00		
	Current smoker	25.7%	33	446	4.4%	(3.8%, 5.2%)	1.09	(0.93, 1.29)	0.29
Hypertension and/or Pre-eclampsia	No HT/P-Ecl	93.3%	90	1,618	4.1%	(2.5%, 6.7%)	1.00		
	HT/P-Ecl	6.7%	6	117	3.5%	(1.6%, 7.7%)	0.86	(0.39, 1.89)	0.71

Table S23: The effect of BMI status at conception and GWG at delivery on the rate of other neonatal complications

Predictor	Predictor groupings	Group % BMI [4]	n	N	% in group with condition [5]	95%CI	IRR [5]	95%CI	P-value
		Outcome: Other neonatal complications							
BMI ($kg.m^2$) at conception [1]	<18.5	3.7%	4	64	2.91%	(1.05%, 8.11%)	1.26	(0.45, 3.50)	0.66
	18.5-25	40.6%	28	704	2.32%	(1.23%, 4.35%)	1.00		
	25-30	27.0%	17	469	2.21%	(1.25%, 3.93%)	0.96	(0.54, 1.70)	0.88
	30-35	14.1%	6	244	1.40%	(0.60%, 3.30%)	0.61	(0.26, 1.43)	0.25
	35+	14.6%	13	254	3.03%	(1.63%, 5.66%)	1.31	(0.70, 2.45)	0.40
Birth GWG versus recommendations [1]	Within	43.5%	37	754	2.32%	(1.23%, 4.35%)	1.00		
	Above	43.6%	21	757	2.04%	(1.20%, 3.47%)	0.88	(0.52, 1.50)	0.64
	Below	12.9%	10	224	2.55%	(1.24%, 5.23%)	1.10	(0.54, 2.26)	0.79
DM screening status & result [2]	GDM excluded	56.0%	24	972	2.32%	(1.23%, 4.35%)	1.00		
	GDM diagnosed	10.5%	10	183	4.47%	(2.22%, 9.00%)	1.93	(0.96, 3.89)	0.065
	GDM not screened	33.4%	34	580	3.67%	(2.15%, 6.26%)	1.59	(0.93, 2.70)	0.090
Whether premature	No prematurity	95.2%	45	1,651	2.32%	(1.23%, 4.35%)	1.00		
	Prem: 32-35 weeks	3.1%	7	53	10.2%	(4.6%, 22.3%)	4.39	(2.00, 9.63)	0.0002
	Prem: <32 weeks	1.8%	16	31	41.1%	(22.8%, 74.1%)	17.75	(9.85, 32.0)	<0.0001
Age of mother (years)	Mother <25	28.2%	19	490	2.32%	(1.23%, 4.35%)	1.00		
	Mother 25-35	59.7%	42	1,035	2.76%	(1.69%, 4.50%)	1.19	(0.73, 1.94)	0.48
	Mother 35+	12.1%	7	210	1.42%	(0.68%, 2.98%)	0.61	(0.29, 1.29)	0.20
SEIFA band [3]	High/middle SEIFA	65.2%	48	1,132	2.32%	(1.23%, 4.35%)	1.00		
	Low SEIFA	34.8%	20	603	1.37%	(0.84%, 2.21%)	0.59	(0.36, 0.95)	0.031
Smoking	Current non-smoker	74.3%	44	1,289	2.32%	(1.23%, 4.35%)	1.00		
	Current smoker	25.7%	24	446	2.57%	(2.09%, 3.16%)	1.11	(0.90, 1.36)	0.32
Hypertension and/or Pre-eclampsia	No HT/P-Ecl	93.3%	61	1,618	2.32%	(1.23%, 4.35%)	1.00		
	HT/P-Ecl	6.7%	7	117	3.82%	(1.84%, 7.92%)	1.65	(0.79, 3.42)	0.18

Table S24: The effect of BMI status at conception and GWG at delivery on the rate of other birth injuries to baby

Predictor	Predictor groupings	Group % BMI [4]	n	N	% in group with condition [5]	95%CI	IRR [5]	95%CI	P-value
				Outcome: Other birth injuries to baby					
BMI (kg.m^2) at conception [1]	<18.5	3.7%	1	64	1.64%	(0.23%, 11.7%)	0.33	(0.05, 2.38)	0.27
	18.5-25	40.6%	41	704	4.93%	(2.81%, 8.65%)	1.00		
	25-30	27.0%	25	469	4.34%	(2.68%, 7.01%)	0.88	(0.54, 1.42)	0.60
	30-35	14.1%	21	244	7.27%	(4.44%, 11.9%)	1.47	(0.90, 2.42)	0.12
	35+	14.6%	12	254	4.33%	(2.22%, 8.44%)	0.88	(0.45, 1.71)	0.70
Birth GWG versus recommendations [1]	Within	43.5%	35	754	4.93%	(2.81%, 8.65%)	1.00		
	Above	43.6%	60	757	8.23%	(5.48%, 12.4%)	1.67	(1.11, 2.51)	0.014
	Below	12.9%	5	224	2.52%	(0.99%, 6.38%)	0.51	(0.20, 1.29)	0.16
DM screening status & result [2]	GDM excluded	56.0%	56	972	4.93%	(2.81%, 8.65%)	1.00		
	GDM diagnosed	10.5%	8	183	4.43%	(2.12%, 9.25%)	0.90	(0.43, 1.87)	0.77
	GDM not screened	33.4%	36	580	5.86%	(3.89%, 8.83%)	1.19	(0.79, 1.79)	0.41
Whether premature	No prematurity	95.2%	96	1,651	4.93%	(2.81%, 8.65%)	1.00		
	Prem: 32-35 weeks	3.1%	2	53	3.37%	(0.87%, 13.0%)	0.68	(0.18, 2.64)	0.58
	Prem: <32 weeks	1.8%	2	31	6.93%	(1.73%, 27.7%)	1.40	(0.35, 5.61)	0.63
Age of mother (years)	Mother <25	28.2%	33	490	4.93%	(2.81%, 8.65%)	1.00		
	Mother 25-35	59.7%	58	1,035	4.07%	(2.71%, 6.11%)	0.83	(0.55, 1.24)	0.36
	Mother 35+	12.1%	9	210	3.21%	(1.58%, 6.53%)	0.65	(0.32, 1.32)	0.24
SEIFA band [3]	High/middle SEIFA	65.2%	63	1,132	4.93%	(2.81%, 8.65%)	1.00		
	Low SEIFA	34.8%	37	603	5.44%	(3.64%, 8.14%)	1.10	(0.74, 1.65)	0.63
Smoking	Current non-smoker	74.3%	78	1,289	4.93%	(2.81%, 8.65%)	1.00		
	Current smoker	25.7%	22	446	4.53%	(3.73%, 5.51%)	0.92	(0.76, 1.12)	0.40
Hypertension and/or Pre-eclampsia	No HT/P-Ecl	93.3%	90	1,618	4.93%	(2.81%, 8.65%)	1.00		
	HT/P-Ecl	6.7%	10	117	6.90%	(3.64%, 13.1%)	1.40	(0.74, 2.65)	0.30
